# Reducing Interface Energy Loss of Perovskite Solar Cells by Molecular Engineering of Hole‐Transporting Materials

**DOI:** 10.1002/anie.202523799

**Published:** 2026-02-11

**Authors:** Guang Shao, Shang‐Gen Yang, Jian Chen, Dian Wang, Jun‐Jie Zhang, Zu‐Kun Zhou, Jing Xiao, Long Jiang, Zhi‐Zheng Wu, Hiroyuki Kanda, Hua Yang, Zeliang Qiu, Ruiyuan Hu, Xingao Li, Ammar Ahmed Khan, Yi Zhang, Jianxing Xia, Mohammad Khaja Nazeeruddin

**Affiliations:** ^1^ School of Chemistry Sun Yat‐sen University Guangzhou Guangdong China; ^2^ Shenzhen Research Institute Sun Yat‐sen University Shenzhen Guangdong China; ^3^ Instrumental Analysis & Research Center Sun Yat‐Sen University Guangzhou Guangdong China; ^4^ Institute of Chemical Sciences and Engineering Ecole Polytechnique Federale de Lausanne (EPFL) Sion Switzerland; ^5^ Institute of High Energy Physics Chinese Academy of Sciences (CAS) Beijing China; ^6^ College of Materials and Chemistry and Chemical Engineering Chengdu University of Technology Chengdu China; ^7^ New Energy Technology Engineering Laboratory of Jiangsu Province, School of Science Nanjing University of Posts and Telecommunications (NJUPT) Nanjing Jiangsu China; ^8^ Department of Physics Lahore University of Management Sciences Lahore Punjab Pakistan; ^9^ Institute of Molecular Plus Tianjin University Tianjin China; ^10^ Department of Mechanical and Energy Engineering College of Engineering Imam Abdulrahman Bin Faisal University Dammam Saudi Arabia; ^11^ School of Integrated Circuits Southeast University Wuxi Jiangsu China

**Keywords:** dopant‐free, energy level alignment, hole‐transporting material, passivation, perovskite solar cell

## Abstract

Numerous novel hole‐transporting materials (HTMs) have been reported in the literature, which play a vital role in enhancing the efficiency and stability of perovskite solar cells (PSCs). However, the PSCs using these HTMs continue to suffer from exciton recombination induced by energy level misalignment and defect states. Herein, an ingenious molecular design for HTMs (**WD03** with triphenylethylene and **WD04** with trithienylethylene) is reported to modulate their energy levels and passivation effectively. The optimal band alignment between **WD03** and perovskite is crucial for enhancing the open‐circuit voltage (*V*_oc_), which minimizes the interface carrier recombination. The theoretical analysis reveals that replacing thiophene with benzene enhances the passivation ability of HTM, resulting in a more substantial passivation effect on the Pb‐cluster defect of perovskite. These factors contribute to a high *V*_oc_ (1.194 V) of **WD03**‐based cell, ranking among the highest values for *n–i–p* PSCs with a normal bandgap perovskite absorber. Moreover, the propeller‐shaped **WD03** strikes an excellent balance between charge transport and film quality. Owing to these advantages, the PSC based on dopant‐free **WD03** with surface modification attains a remarkable efficiency of 23.66% and the PSC based on doped **WD03** reaches an exceptional efficiency of 25.79%. Following the substitution of trithienylethylene with triphenylethylene, the **WD03**‐based cell exhibits enhanced stability compared to the cell based on **WD04**. This work emphasizes the significance of molecular engineering of HTMs in regulating energy level and passivation ability, which are crucial for achieving high *V*_oc_ and stability in PSCs.

## Graphical abstract

This study reports a new hole‐transporting material (**WD03**) designed via molecular engineering to regulate defect passivation and energy‐level alignment in perovskite solar cells (PSCs). The propeller‐shaped structure ensures balanced charge transport and film quality. Consequently, **WD03**‐based devices deliver a high open‐circuit voltage of 1.194 V under doped conditions and achieve an efficiency of 23.66% in dopant‐free devices with surface modification, underscoring the importance of molecular design for high‐performance and stable PSCs.

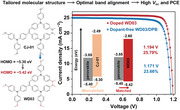

## 1 Introduction

Over the past decade, organic–inorganic hybrid perovskite solar cells (PSCs) have undergone rapid development, emerging as one of the most promising photovoltaic technologies, with a remarkable power conversion efficiency (PCE) of more than 27% [1]. This achievement is partially attributed to the persistent exploitation of hole‐transporting materials (HTMs), which serve multiple functions within the cells, such as conducting holes, blocking electrons, resisting humidity, and passivating defects [2, 3, 4]. Plenty of organic small molecule‐based HTMs have been synthesized and employed in PSCs. According to the main skeleton or functional groups, these HTMs can be categorized as derivatives of fluorene [5], thiophene [6], carbazole [7], helicene [8], sacridine [9], azulene [10], naphthalene [11], and so forth. Currently, the most extensively used HTM is 2,2′,7,7′‐tetrakis(*N*,*N*‐di‐*p*‐methoxyphenylamine)‐9,9′‐spirobifluorene (spiro‐OMeTAD) due to its superior performance in negative–intrinsic–positive (*n–i–p*) PSCs [12]. However, the hole mobility (*μ*) of pristine spiro‐OMeTAD is relatively low (∼ 10^−5^ cm^2^ V^−1^ s^−1^) on account of weak intermolecular interactions stemming from its inherent orthogonal molecular conformation [13]. Thus, spiro‐OMeTAD heavily relies on the dopants for high hole mobility, for example, lithium bis(trifluoromethylsulfonyl)imide (Li‐TFSI) and 4‐*tert*‐butylpyridine (*t*‐BP) [14]. Unfortunately, the hygroscopicity of Li‐TFSI, the volatility of *t*‐BP, and their tendency to diffuse seriously impair the device stability. Besides, the doping processes bring about additional costs, complications, and issues of reproducibility in device fabrication [15]. Therefore, developing dopant‐free HTMs is a wise strategy to avoid the aforementioned problems and facilitate the eventual commercialization of PSCs.

Generally, there are two primary approaches for designing dopant‐free HTMs. The first option is to incorporate strong intramolecular interactions to form a self‐planarized backbone, thereby enhancing intermolecular *π–π* interactions and the corresponding charge transport. However, a significant drawback of these materials is the formation of coarse films, which arises from their strong propensity to crystallize during the spin‐coating process. The other one is to construct an electron donor–acceptor (D–A) structure. The D–A‐type molecules possess improved hole mobility induced by strengthened intermolecular packing through intense dipole–dipole interactions. Additionally, they exhibit zwitterionic resonance in their ground state due to robust intramolecular charge transfer, potentially enabling an induced doping effect without dopants. Nevertheless, achieving suitable zwitterionic properties alongside satisfactory hole mobility is challenging, as most D–A‐type molecules conform to the Bässler model, which posits that the intrinsic dipolarity of a molecule is detrimental to its charge transport capabilities [16, 17, 18, 19]. Additionally, the performance of PSCs is restricted by trap‐assisted non‐radiative recombination occurring at the perovskite/HTM interface [20]. The under‐coordinated Pb^2+^ ions serve as a primary source of trap states, acting as non‐radiative recombination centers for charge carriers [21]. The vacant *6p* orbitals of unsaturated Pb^2+^ ions can accept lone pairs of electrons from electron‐rich Lewis bases, such as carbonyl groups and functional groups containing S or N atoms, to form coordination bonds, that is, passivation. Previous studies have shown that sulfur‐containing conjugated frameworks, such as methylthio‐ or thiophene‐based HTMs, can coordinate with undercoordinated Pb^2+^ ions at the perovskite surface through Pb–S interactions, thereby suppressing recombination and promoting charge transport [22]. Accordingly, incorporating passivation groups into HTMs represents an effective strategy to enhance the performance of PSCs [23].

Despite numerous HTMs featuring improvements in hole mobility [24], dopants [25], trap state passivation [26], and energy level regulation [27], interface trap recombination and energy loss remain significant problems. These challenges contribute to low open‐circuit voltage (*V*_oc_) and instability of the PSCs based on these HTMs, regardless of whether they are doped or dopant‐free. It is worth noting that most HTMs exhibit higher highest occupied molecular orbital (HOMO) levels than the valence band maximum (VBM) of perovskite (Δ*E* > 0.1 eV), resulting in a considerable energy loss at the interface. However, there is very limited literature on addressing the conditions for nearly aligned energy levels, which theoretically minimizes the energy loss.

Here, we further optimize the molecular structure of the previously reported HTM (**CJ‐01**). Our findings demonstrate that this triphenylethylene‐based HTM enables the construction of a three‐dimensional charge transport network without compromising its film‐forming capability [28]. Due to the larger atomic radius and lower electronegativity compared to the O atom, the S atom exhibits a greater tendency for its valence electrons to coordinate with the Pb^2+^ ion, resulting in a more substantial passivation effect. In the meantime, the weaker electron‐donating capacity of the methylthio unit relative to the methoxy unit lowers the HOMO level of HTM, matching it with the VBM of the perovskite and thus reducing energy loss [29]. Consequently, to maximize the potential of triarylethylene‐based molecules, the methoxy units in **CJ‐01** were replaced by the methylthio units to create **WD03**. Given the prevalence of electron‐rich thiophene rings as HTM building blocks for energy levels and charge transport modulation, the triphenylethylene in **WD03** was substituted with the trithienylethylene to generate **WD04** [30]. Their physicochemical properties were examined using both experimental and theoretical methods, and their efficacy in PSCs was also evaluated. Our research aims to minimize the interface energy loss in the cells by optimizing the energy level and passivation ability of HTMs.

## 2 Results and Discussion

### 2.1 Molecular Properties

**FIGURE 1:**
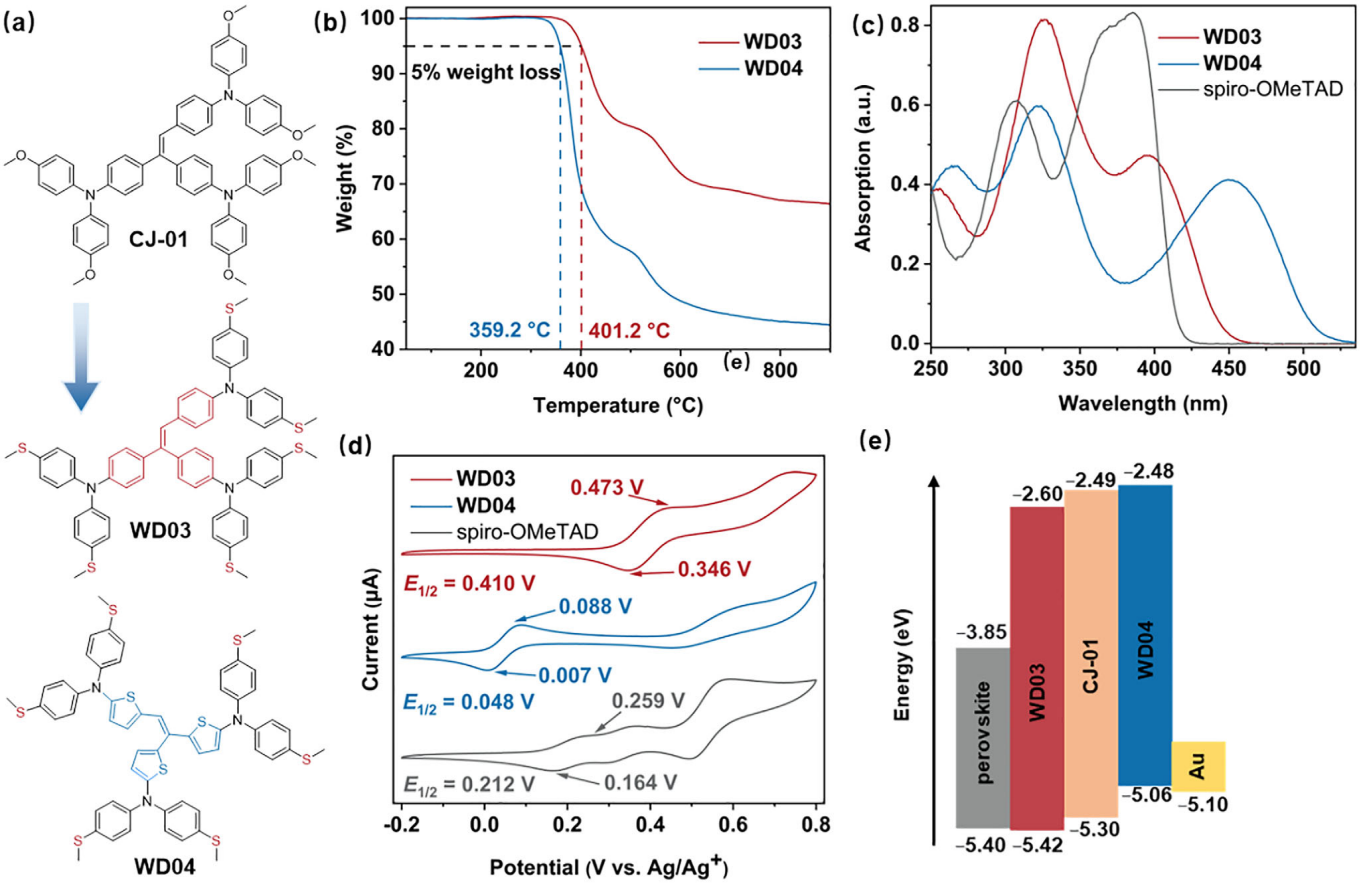
(a) Molecular structures of **CJ‐01**, **WD03**, and **WD04**. (b) TGA of **WD03** and **WD04**. (c) UV–Vis absorption spectra of **WD03**, **WD04**, and spiro‐OMeTAD in CH_2_Cl_2_ (1.0 × 10^−5^ M). (d) Cyclic voltammograms of **WD03**, **WD04**, and spiro‐OMeTAD in CH_2_Cl_2_ with TBAPF_6_ (1.0 × 10^−4^ M, vs. Ag/Ag^+^). (e) Energy level alignment of components in PSC.

The structures of the new HTMs (**WD03** and **WD04**) are illustrated in Figure 1a. The synthesis and structural verification are described in the Supporting Information (Scheme S1 and Figures S1–S14). The core brominated triphenylethylene (**6**) was synthesized according to the previous work [28]. Similarly, another core brominated trithienylethylene (**5**) was constructed through Wittig–Horner reaction. Finally, the target HTMs were obtained through Buchwald–Hartwig coupling of their respective cores with 4,4′‐dimethylthiodiphenylamine (**1**). The thermal properties of **WD03** and **WD04** were investigated using thermogravimetric analysis (TGA) and differential scanning calorimetry measurements (Figures 1b and S15). The decomposition temperature (*T*_d_) of **WD03** at a 5% weight loss is 401.2°C, which is higher than that of **WD04** (359.2°C). The relative instability of **WD04** results from the fact that the thiophene ring is less stable than the benzene ring. In Figure S15, the first heating curves show that **WD03** and **WD04** melt at 146.2 and 89.3°C, respectively, confirming their crystalline nature. During the second heating, the glass‐transition temperatures (*T*_g_) of **WD03** and **WD04** are found to be 106.1 and 79.7°C, respectively.

The UV–Vis absorption spectra of **WD03** and **WD04** in CH_2_Cl_2_ (Figure 1c and Table 1) reveal three distinct absorption bands within the 250–535 nm range. As anticipated, the absorption spectrum of **WD04** exhibits a red shift relative to that of **WD03**, which can be attributed to the strong electron‐donating ability of the thienyl groups [32]. In comparison, the UV–Vis absorption spectra of the films (Figure S16 and Table S1) reveal slightly red‐shifted absorption bands for both **WD03** and **WD04**, indicating their intermolecular interactions in the solid state [33]. In contrast, the maximum absorption peaks of spiro‐OMeTAD in film are almost identical to those in CH_2_Cl_2_, indicating a lack of aggregation and *π–π* stacking in the solid state [34, 35]. From the intersection of the normalized UV–Vis absorption and emission spectra (439, 481, and 407 nm, for **WD03**, **WD04**, and spiro‐OMeTAD, respectively, Figures S17–S20), optical band gaps (*E*_gap_ = 1240/*λ*) are calculated to be 2.82, 2.58, and 3.05 eV, respectively.

**TABLE 1:** Photophysical, electrochemical, and thermal properties of HTMs.

HTM	*λ*_abs_ (nm)^a^	HOMO (eV)	LUMO (eV)^b^	*E*_gap_ (eV)^c^	*T*_d_ (°C)^d^	*T*_g_ (°C)	*μ* (cm^2^ V^−1^ S^−1^)^f^
**WD03**	256, 326 (max), 395	−5.42	−2.60	2.82	401.2	106.1	4.78 × 10^−5^
**WD04**	264, 321 (max), 450	−5.06	−2.48	2.58	359.2	79.7	1.44 × 10^−4^
spiro‐OMeTAD	308, 385 (max)	−5.22	−2.17	3.05	417.0^e^	126.0^e^	2.90 × 10^−5^

^a^ In CH_2_Cl_2_.

^b^ *E*_LUMO_ = *E*_HOMO_ + *E*_gap_.

^c^ Calculated from the intersection of normalized absorption and emission spectra.

^d^ 5% weight loss.

^e^ Reference [31].

^f^ Undoped.

Cyclic voltammetry was adopted to estimate the energy levels of HTMs (Figure 1d). First, *E*_1/2_ (average value of the first redox peaks) of HTM is compared with that of spiro‐OMeTAD, that is, Δ*E*_1/2_ = *E*_1/2 (HTM)_ − *E*_1/2 (spiro‐OMeTAD)_. Subsequently, the HOMO level of HTM is determined from the equation: *E*_HOMO (HTM)_ = *E*_HOMO (spiro‐OMeTAD)_ − Δ*E*_1/2_. Here, the HOMO level of spiro‐OMeTAD is −5.22 eV [36]. Finally, the lowest unoccupied molecular orbital (LUMO) level is calculated using the equation: *E*_LUMO_ = *E*_HOMO_ + *E*_gap_. The energy levels of HTMs are listed in Table 1. The close alignment between the HOMO level of **WD03** and the VBM of perovskite may lead to a higher *V*_oc_ by reducing energy loss at the interface (Figure 1e) [37].

Quantum chemical calculations were carried out by employing density functional theory (DFT) with B3LYP exchange‐correlation functional and 6–31G basis set (Tables S2–S4). Owing to the steric hindrance of adjacent groups, the optimized configurations of **WD03** and **WD04** assume a propeller‐like shape. The HOMOs of **WD03** and **WD04** are distributed throughout the entire molecules, while their LUMOs are dominantly located in the cores. This means that the electrons transfer from the peripheral groups to the central part upon excitation. Their electrostatic surface potential maps reveal that negative charges are concentrated on the S atoms, which potentially act as Lewis bases to passivate the defects of perovskite [38]. The *μ* of pristine HTMs was measured through the space‐charge‐limited current (SCLC) method in a hole‐only device with an architecture of FTO/PEDOT:PSS/HTM/Au (Table 1 and Figure S21). In summary, **WD04** (1.44 × 10^−4^ cm^2^ V^−1^ S^−1^) exhibits the highest hole mobility owing to its thiophene‐rich core, which enables stronger charge delocalization and intermolecular electronic coupling compared with **WD03** (4.78 × 10^−5^ cm^2^ V^−1^ S^−1^) and spiro‐OMeTAD (2.90 × 10^−5^ cm^2^ V^−1^ S^−1^). However, despite its enhanced mobility, the unfavorable energy‐level alignment of **WD04** leads to inferior device performance compared to **WD03**.

**FIGURE 2:**
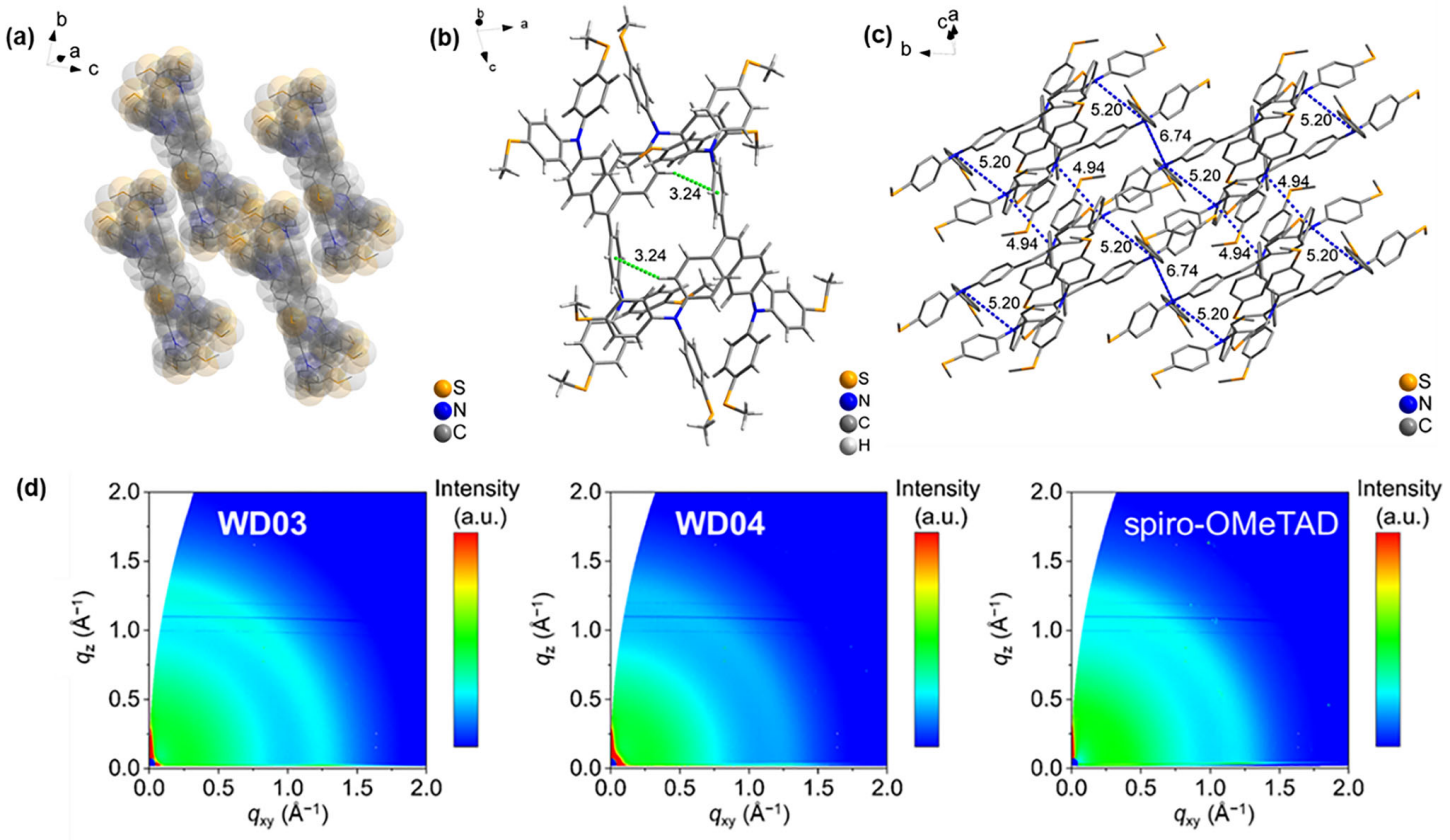
(a) Molecular packing of **WD03** (H atoms were omitted for clarity). (b) CH···*π* interactions in **WD03** single crystal (green dashed lines; unit: Å). (c) N–N distances between the layers of **WD03** molecules (blue dashed line; unit: Å; H atoms were omitted for clarity.). (d) GIWAXS of **WD03**, **WD04**, and spiro‐OMeTAD on a silicon wafer.

Single crystals of **WD03** were grown via the slow evaporation of a CH_2_Cl_2_/MeOH solution (CCDC: 2469846; space group: *P* $\bar{1}$, Table S5). The molecular configuration, packing, and intermolecular interactions of **WD03** were elucidated by single‐crystal x‐ray diffraction. The three benzene rings of triphenylethylene tilt at 20.05°, 66.84°, and 27.40° against the plane of the double bond, presenting a propeller shape (Figures S22–S24 and Video S1). The unit cell contains two molecules that are arranged in an interlaced manner when viewed along the *a* axis (Figure S25 and Video S2). The molecular packing diagram reveals that the molecules adopt a slipped and interlaced arrangement, forming a twisted structure (Figure 2a,b). Because of the twisted configuration, abundant CH···*π*, S···S, and CH···S interactions exist in the crystal, which bind the molecules together and facilitate charge transport (Figures 2b and S26). In organic semiconductors, charge transport primarily occurs through a sequence of incoherent hopping events rather than through band‐like motion [28]. In **WD03**, the holes are primarily localized on the triphenylamine units, which serve as the dominant redox centers. Consequently, hole transport in the **WD03** film can be conceptualized as thermally activated hopping between the triphenylamine sites of adjacent molecules. Accordingly, the distances between triphenylamine units in adjacent molecules are crucial to charge transport. The N–N distances within a layer of molecules are 8.36, 10.09, 10.99, 11.22, and 12.51 Å, respectively (Figure S27); and the N–N distances of layer‐to‐layer molecules are 4.94, 5.20, and 6.74 Å, respectively (Figure 2c and Video S3). The numerous and tightly packed triphenylamine units are favorable for hole‐hopping.

Grazing incidence wide‐angle x‐ray scattering (GIWAXS) measurements were performed to explore the molecular stacking and orientation of HTMs in films (Figure 2d). **WD03** exhibits pronounced and concentrated scattering intensities at *q* ≈ 0.75 and 1.25  Å^−1^, indicating a high degree of crystallinity. The diffraction ring at 1.25  Å^−1^ is broad in the out‐of‐plane direction and narrow in the in‐plane direction, indicating a preferentially horizontal orientation in the layered stacking. In contrast, **WD04** shows partial crystallinity and random orientation, as evidenced by broader and less intense diffraction signals. Spiro‐OMeTAD displays a diffuse scattering pattern devoid of distinct diffraction features, implying its amorphous structure. A higher proportion of face on‐oriented crystallites on the perovskite surface is beneficial for improving the hole extraction efficiency of **WD03**.

### 2.2 Trap States Passivation

**FIGURE 3:**
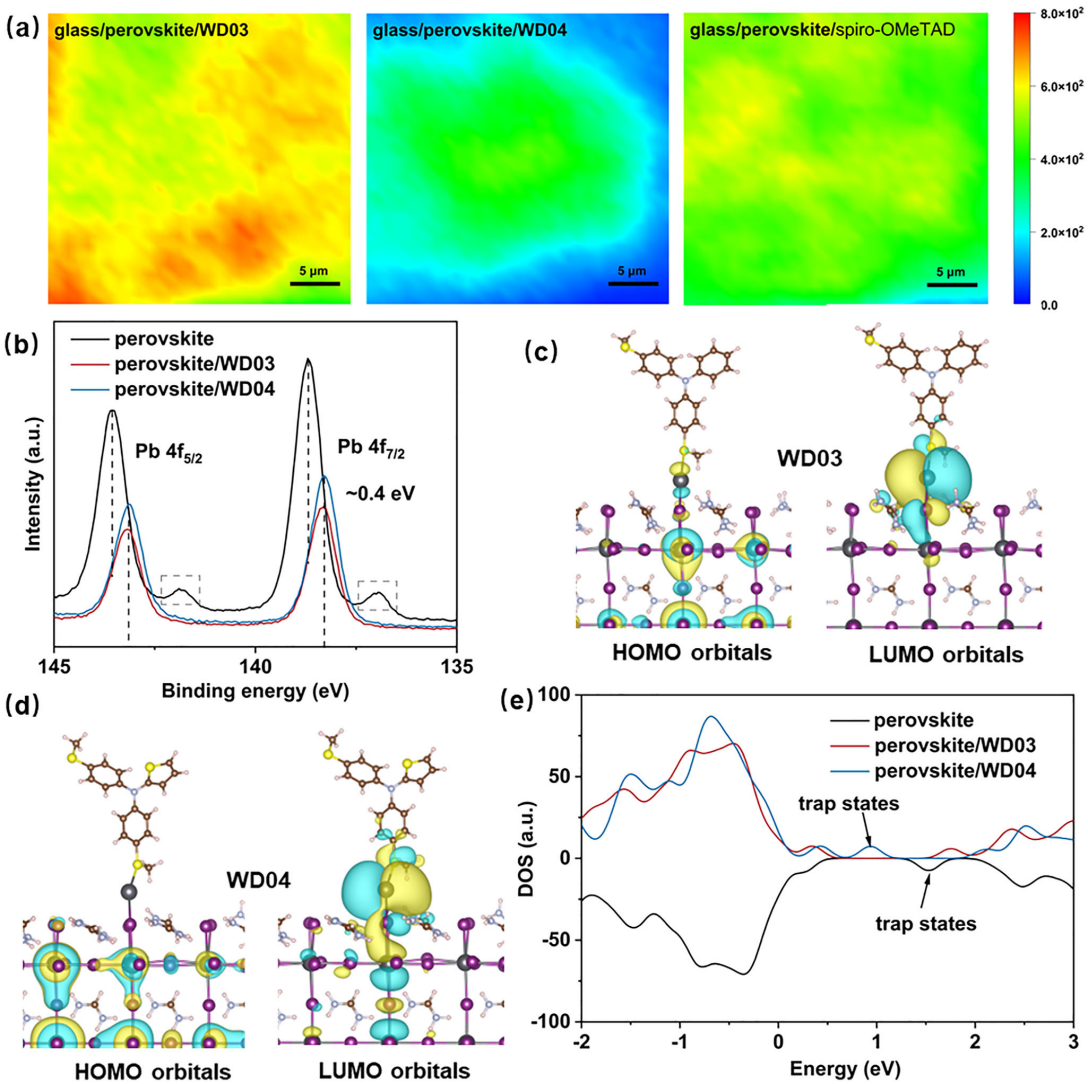
(a) PL mapping images of the samples based on glass/perovskite/HTM. (b) XPS spectra of perovskite, perovskite**/WD03**, and perovskite**/WD04**. HOMO and LUMO orbitals of partial (c) **WD03** and (d) **WD04** adsorbed on the Pb‐dimer‐based perovskite (001) surface (brown: C; pink: H; light blue: N; yellow: S; purple: I; black: Pb). (e) DOS of perovskite, perovskite**/WD03**, and perovskite**/WD04**.

The photoluminescence (PL) mapping was employed to investigate the passivation effect of HTMs on perovskite layers (Figure 3a). The samples were structured as glass/perovskite/HTM (scale: 25 µm × 25 µm). The concentrations of the HTM solutions were set at 1.5 mg mL^−1^ to produce a non‐transport structure. The higher PL intensity observed in the **WD03**‐based sample compared to the **WD04**‐ and spiro‐OMeTAD‐based ones suggests that **WD03** has a superior defect passivation ability, thereby resulting in lower surface carrier recombination. Despite possessing a greater number of S atoms than **WD03**, **WD04** does not show any improvement in passivation capability, suggesting that the factors beyond S atom quantity dominate the passivation process. The twisted conformation of **WD04** hinders the S atoms in thiophene rings from interacting with Pb^2+^ ions, likely explaining this phenomenon [39].

X‐ray photoelectron spectroscopy (XPS) measurements were conducted to clarify the interactions between HTMs and perovskite (Figure 3b). The bare perovskite shows two dominant peaks belonging to fully coordinated Pb^2+^ ions (138.7 eV for *4f_7/2_* and 143.6 eV for *4f_5/2_*) and two smaller peaks corresponding to unsaturated Pb^2+^ ions (137.0 and 141.9 eV) [40]. After depositing HTMs on the perovskite, the Pb *4f* peaks shift ∼ 0.4 eV toward lower binding energy and the two smaller peaks disappear, manifesting the coordination and passivation effect of HTMs on the perovskite (Pb–S bonding) [41]. Moreover, the I *3d* signals from the perovskite/HTM bilayers shift toward lower binding energy relative to those from the perovskite film, likely because Pb^2+^ ions can accept lone pairs of electrons from HTMs, thereby disturbing the electrostatic interactions between Pb^2+^ and I^−^ ions (Figure S28) [42].

To further probe the passivation mechanism, DFT calculations were employed to study the bonding interactions between the units of **WD03**/**WD04** and the Pb‐cluster‐based perovskite (001) surface. Both the optimized geometries covered with HOMOs and LUMOs are exhibited in Figure 3c,d. From the adsorption models, it is evident that both units of **WD03** and **WD04** directly bond to the Pb‐cluster through S atoms, which is consistent with the findings from the XPS results. The less occupied LUMO around the Pb‐cluster in **WD03** relative to **WD04** signifies a greater electron donation from **WD03** to the Pb‐cluster. The results theoretically indicate a higher passivation ability of **WD03**. To elucidate the changes in electronic state induced by passivation, the density of states (DOS) of perovskite defects without and with **WD03**/**WD04** interactions were analyzed (Figure 3e). Before passivation, the trap states are present within the band gap of the perovskite, typically functioning as centers for charge recombination. When the **WD03** unit interacts with the Pb‐cluster, the trap states are eliminated, indicating the complete passivation of surface trap states in the perovskite. However, the **WD04** unit merely shifts the trap states to the valence band, converting the deep trap states into the shallow ones. It reduces carrier recombination, nevertheless, less effectively than the **WD03** unit, which is also consistent with the experimental results.

### 2.3 Film Morphologies and Carrier Transport

**FIGURE 4:**
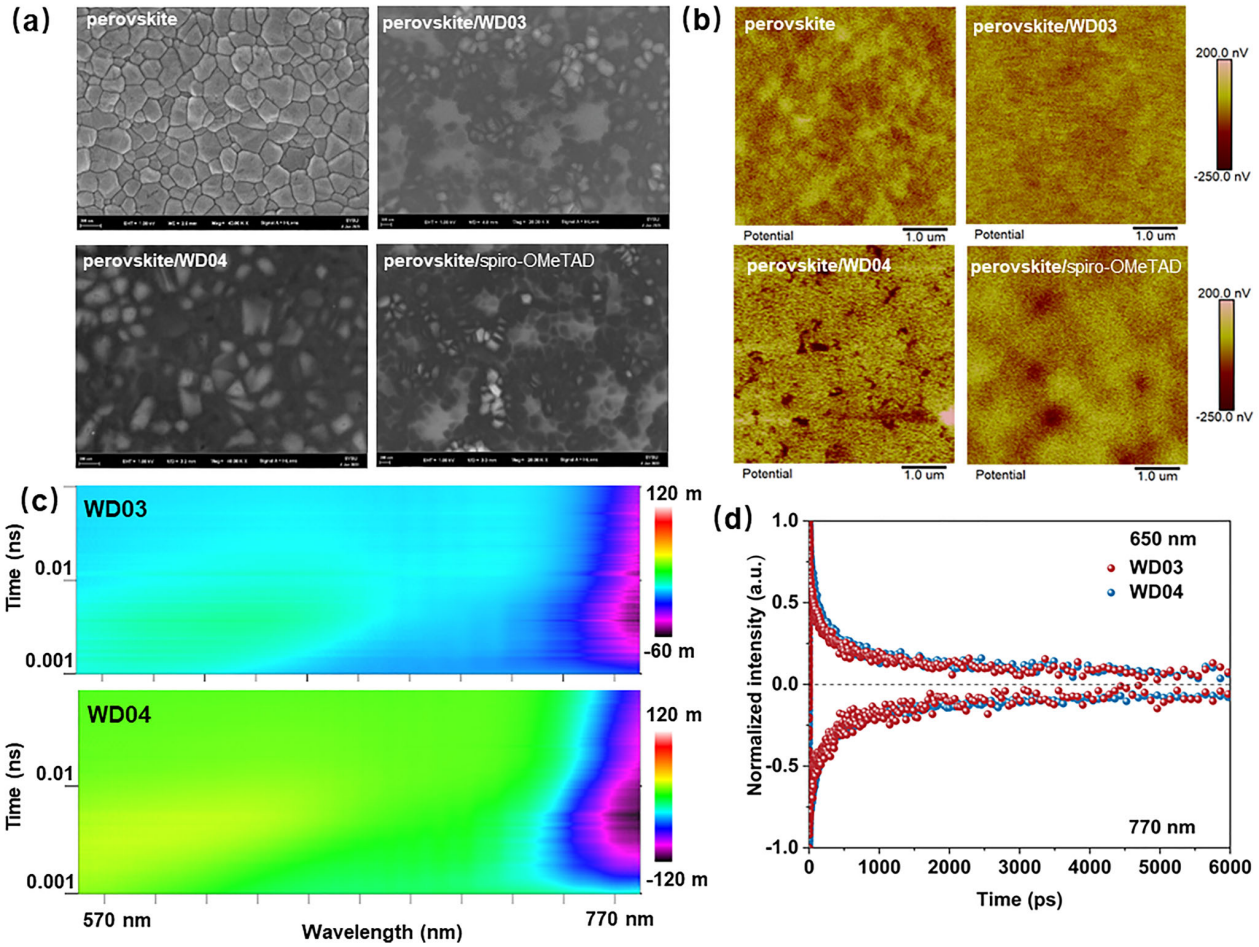
(a) SEM top‐view images and (b) KPFM images of perovskite and HTMs spin‐coated on perovskite. (c) Femtosecond transient absorption (fs‐TA) spectra of glass/perovskite/HTM films. (d) Normalized kinetic traces of photobleaching probed at 650 and 770 nm.

The film‐forming ability of undoped HTMs was assessed by scanning electron microscope (SEM) and atomic force microscopy (AFM) (Figures 4a, S29, and S30). The perovskite film is polycrystalline and rough with a surface roughness (*R*_q_) of 31.9 nm. After the spin‐coating of HTMs at the concentrations identical to those used in device fabrication, the grain boundaries of perovskite became blurred, indicating the complete coverage of HTM films. **WD04**, owing to the inferior film‐forming property, displays evident voids, thereby increasing the roughness (*R*_q_ = 27.5 nm). In contrast, both **WD03** and spiro‐OMeTAD exhibit smoother morphologies (*R*_q_ = 12.2 and 11.1 nm, respectively).

Kelvin probe force microscopy (KPFM) experiments were carried out to investigate the surface potentials of perovskite film and perovskite/HTM films (Figure 4b). The perovskite film exhibits evident potential fluctuations, suggesting an inhomogeneous potential distribution. The introduction of **WD03** yields a more uniform surface potential, implying that **WD03** effectively passivates surface defects of perovskite, thereby enabling a homogeneous charge extraction interface. In the perovskite/**WD04** sample, the image clearly shows distinct holes on the surface, exposing the underlying perovskite layer. This observation indicates the inadequate film‐forming capability of **WD04**. The perovskite/spiro‐OMeTAD sample exhibits localized regions of low surface potential, which are attributed to the prominent perovskite grains protruding through the HTM layer.

The fs‐TA spectra were recorded to analyze the charge dynamics at the perovskite/HTM interfaces (Figures 4c and S31). Both samples display a ground‐state bleaching (GSB) peak near 770 nm, coinciding with the absorption edge of perovskite. The weaker GSB signal of perovskite/**WD03** film, compared to perovskite/**WD04** film, indicates a faster extraction of holes [43]. The decay of excited state absorption bands at 650 nm and GSB peaks was fitted using a bi‐exponential decay function (Figures 4d, S32, and Table S6). The rapid process (*τ*_1_) is attributed to the extraction of holes [44]. The average carrier lifetimes of perovskite/**WD03** film at 650 and 770 nm are 366.52 and 448.86 ps, respectively, indicating a lifetime range from 366.52 to 448.86 ps. In contrast, the perovskite/**WD04** film shows longer lifetimes (546.30 and 551.10 ps). The shorter *τ*_1_ and average decay time of perovskite/**WD03** film suggest faster charge extraction and reduced recombination loss.

### 2.4 Photovoltaic Performance

**FIGURE 5:**
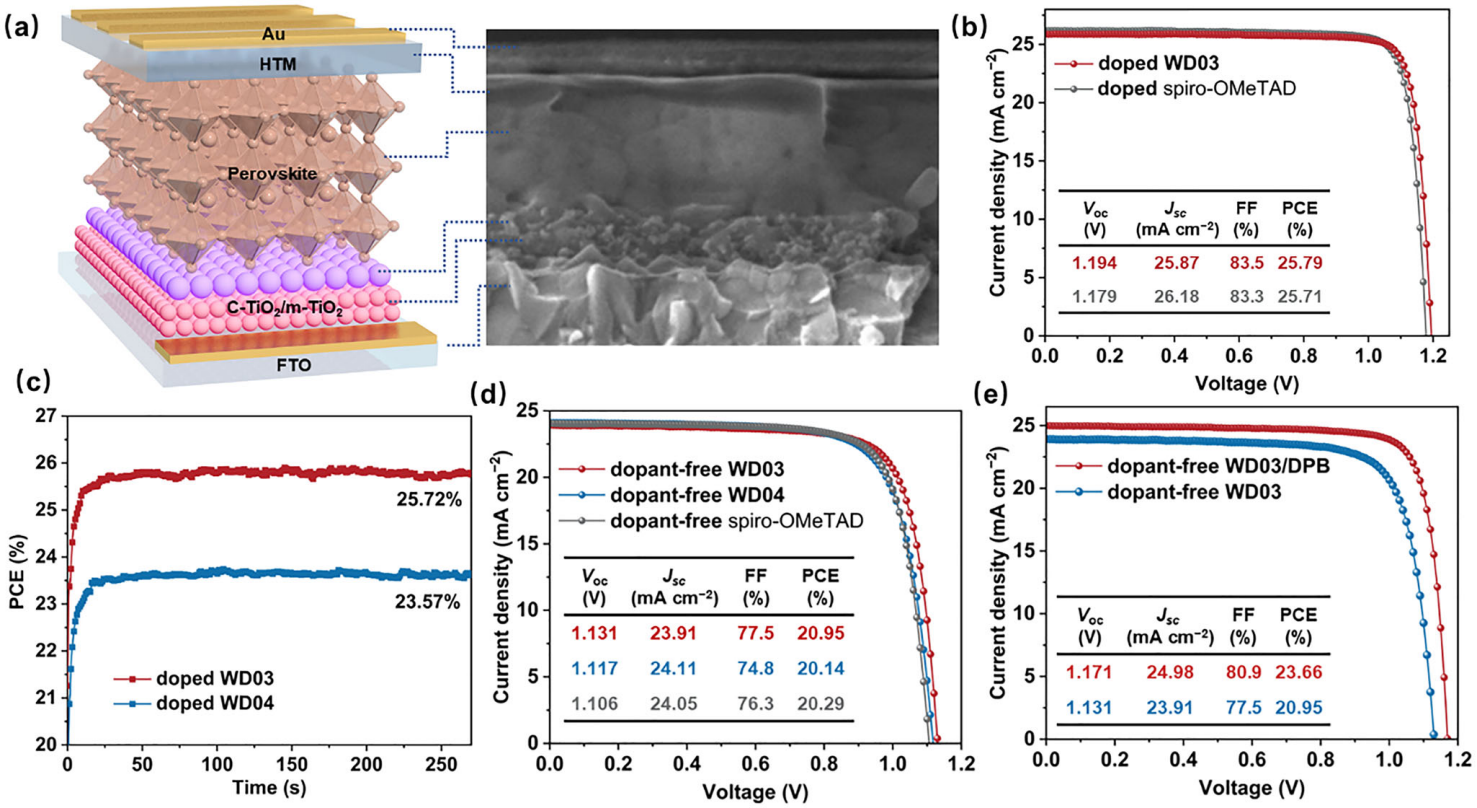
(a) Schematic illustration of device structure and corresponding SEM cross‐sectional image. (b) *J–V* curves of PSCs based on doped **WD03** and spiro‐OMeTAD. (c) Stabilized power output at MPP of PSCs based on doped **WD03** and **WD04**. (d) *J–V* curves of PSCs based on dopant‐free **WD03**, **WD04**, and spiro‐OMeTAD. (e) *J–V* curves of PSCs based on dopant‐free **WD03** and **WD03**/DPB.

PSCs based on a structure of FTO/compact TiO_2_ (c‐TiO_2_)/mesoporous TiO_2_ (m‐TiO_2_)/perovskite/HTM/Au were fabricated to evaluate the effectiveness of HTMs (Figure 5a). The perovskite film is dense, with a thickness of ∼ 520 nm and a grain size distribution ranging from 100 to 600 nm. The HTM film completely covers the perovskite layer, with a thickness of ∼ 60 nm. These features facilitate the realization of high‐efficiency PSCs. The current density–voltage (*J–V*) curves of champion devices based on doped HTMs are displayed in Figures 5b and S33, and the relevant parameters are compiled in Table S7. The cell using doped **WD03** yields a PCE of 25.79%, which is comparable to that of the doped spiro‐OMeTAD‐based cell (25.71%) and higher than that of the doped **WD04**‐based cell (23.67%). **WD03** possesses a deeper HOMO level than that of **WD04**, leading to a higher *V*_oc_ (1.194 vs. 1.145 V), but a lower short‐circuit current density (*J*_sc_) (25.87 vs. 26.01 mA cm^−2^). It is worth mentioning that 1.194 V ranks among the highest values for *n–i–p* PSCs. The current density integrated from the incident photon‐to‐current conversion efficiency spectrum of the doped **WD03**‐based cell is 25.19 mA cm^−2^, close to its *J*_sc_ (Figure S34). The stabilized PCEs at the maximum power point (MPP) of devices based on doped **WD03** and **WD04** are 25.72% and 23.57%, respectively, approximating the PCEs derived from the *J–V* curves (Figure 5c).

**FIGURE 6:**
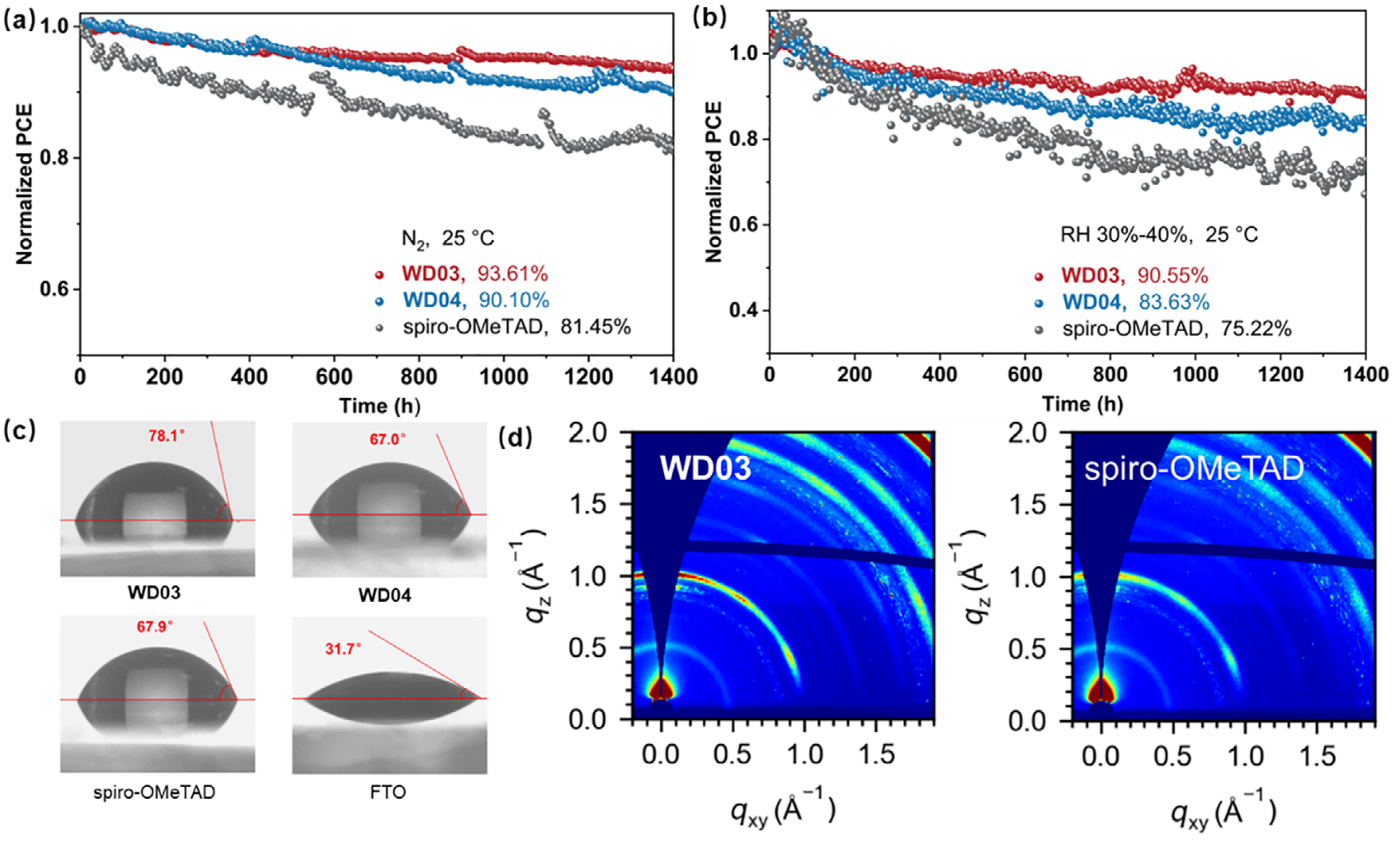
Continuous MPP tracking of cells containing doped HTMs under conditions of (a) N_2_, 25°C and (b) air, 30%–40% RH, 25°C. (c) Water contact angles of FTO glass and HTM films on FTO glass. (d) GIWAXS of aged devices (30 days, in air, RH 30%–40%, and 25°C) with doped **WD03** and spiro‐OMeTAD.

Dopant‐free HTM‐based cells were also fabricated (Figure 5d and Table S8). The optimal cell incorporating undoped **WD03** delivers a PCE of 20.95% with a *J*_sc_ of 23.91 mA cm^−2^, a *V*_oc_ of 1.131 V, and an FF of 77.5%. These performances outperform those from the cells employing undoped **WD04** (PCE = 20.14%, *J*_sc_ = 24.11 mA cm^−2^, *V*_oc_ 1.117 V, and FF = 74.8%) and spiro‐OMeTAD (PCE = 20.29%, *J*_sc_ = 24.05 mA cm^−2^, *V*_oc_ 1.106 V, and FF = 76.3%). However, the results are inferior to those of the doped cells, which arise from the mismatch of energy levels between the undoped HTMs and the Au electrode. Inspired by prior work, where *N*,*N*‐dimethylanilinium tetrakis(pentafluorophenyl)borate (DPB) can elevate the surface HOMO level of HTM to match the energy level of the Au electrode, we applied DPB to modify undoped **WD03** [45]. As a result of treatment, the PCE of the cell using dopant‐free **WD03** is raised to 23.66%, accompanied by the enhanced parameters (*J*_sc_ = 24.98 mA cm^−2^, *V*_oc_ 1.171 V, and FF = 80.9%) (Figure 5e and Table S8). The MPP tracking of devices containing doped HTMs is presented in Figure 6a,b. In an N_2_ atmosphere, **WD03**‐, **WD04**‐, and spiro‐OMeTAD‐based cells preserve 93.61%, 90.10%, and 81.45% of the initial PCE after 1400 h of continuous operation, respectively. Under an ambient environment with 30%–40% relative humidity (RH), the PCE retention drops to 90.55%, 83.63%, and 75.22%, respectively, primarily due to the moisture‐driven decomposition of perovskite. The thermal stability test at MPP was conducted at 65°C under an N_2_ atmosphere (Figure S35). After 550 h, the **WD03**‐ and **WD04**‐based devices retains 92.89% and 91.72% of their initial PCE, respectively. In contrast, the spiro‐OMeTAD‐based device exhibits a retention of only 83.68%. The findings underscore the exceptional stability of the **WD03**‐based device.

Water contact angles of HTMs spin‐coated on FTO glass were measured to analyze their hydrophobicity (Figure 6c). The contact angles of **WD03**, **WD04**, and spiro‐OMeTAD are 78.1°, 67.0°, and 67.9°, respectively, signifying the highest hydrophobicity of **WD03**. Meanwhile, **WD04** and spiro‐OMeTAD demonstrate relatively poor yet similar hydrophobicity. A hydrophobic HTM film effectively shields the perovskite from humidity, contributing to its long‐term stability. The moisture‐resistance ability of doped HTMs was assessed by GIWAXS tests on devices aged for 30 days in air with 30%–40% RH (Figure 6d). The **WD03**‐based cell exhibits a stronger diffraction peak corresponding to the (001) plane of perovskite at *q* = 1.00 Å^−1^ compared to the spiro‐OMeTAD‐based one, indicating a more intact perovskite structure in the former.

## 3 Conclusions

In summary, two HTMs (**WD03** and **WD04**) comprising a triphenylethylene or trithienylethylene core and *p*‐methylthiodiphenylamine moieties were constructed. Benefiting from the methylthio groups, **WD03** exhibits robust passivation capability and slight energy offset. These factors synergistically contribute to the outstanding performance of the **WD03**‐based cell in the doped state (PCE = 25.79% and *V*_oc_ = 1.194 V), which is superior to that of the cells with **WD04** and spiro‐OMeTAD, respectively. The **WD03**‐based cell is the most stable under both N_2_ and ambient (30%–40% RH) conditions owing to its highest hydrophobicity. The propeller‐shaped geometry of **WD03** induces proper crystallinity and ordered molecular stacking, reaching an effective balance between charge transport capability and film‐forming ability. As a result, the dopant‐free **WD03**‐based cell accomplishes a PCE of 23.66% with DPB treatment, despite its moderate hole mobility. This study opens a promising avenue for exploiting HTMs with passivation effect and small energy offset to endow PSCs with high *V*_oc_ and long‐term stability concurrently.

## Author Contributions

G. S. and S.‐G. Y. fabricated the cells and wrote the manuscript. J. C., D. W., and J.‐J. Z. synthesized the HTMs and measured the photophysical, electrochemical, and thermal properties. Z.‐K. Z. and J. X. (Jing Xiao) conducted the SEM, AFM, and KPFM measurements. L. J. and Z.‐Z. W. implemented the XPS, x‐ray crystallography, and water contact angle measurements. H. K., H. Y., Z.‐L. Q., R.‐Y. H., X.‐A. L., and A. A. K. carried out the SCLC, GIWAXS, PL mapping, and fs‐TA tests. J. X. (Jianxing Xia) performed the DFT theory, put forward the DPB strategy, and wrote the manuscript. Y. Z. revised the manuscript. J. X. (Jianxing Xia), G. S, and M. K. N. supervised the project. All authors discussed and revised the manuscript.

## Conflicts of Interest

The authors declare no conflicts of interest.

## Supporting information




**Supporting File 1**: anie71480‐sup‐0001‐SuppMat.docx.

**Supporting File 2**: anie71480‐sup‐0002‐VideoS1.mp4.

**Supporting File 3**: anie71480‐sup‐0003‐VideoS2.mp4.

**Supporting File 4**: anie71480‐sup‐0004‐VideoS3.mp4.

## Data Availability

The data that support the findings of this study are available in the Supporting Information of this article.
