# Supplementary material for: Reducing Interface Energy Loss of Perovskite Solar Cells by Molecular Engineering of Hole‐Transporting Materials
Source: Angew Chem Int Ed Engl. 2026 Feb 11;65(13):e23799. doi: 10.1002/anie.202523799 (PMC13007580; doi:10.1002/anie.202523799)
Supplement: Supplementary file 1 — Supporting File 1: anie71480‐sup‐0001‐SuppMat.docx. [file ANIE-65-e23799-s001.docx]

**Supporting Information**

**Reducing Interface Energy Loss of Perovskite Solar Cells by Molecular Engineering of Hole-Transporting Materials**

Guang Shao,^1,4*^ Shang-Gen Yang,^1^ Jian Chen,^1,4^ Dian Wang,^1,4^ Jun-Jie Zhang,^1^ Zu-Kun Zhou,^1^ Jing Xiao,^1,4^ Long Jiang,^5^ Zhi-Zheng Wu,^1^ Hiroyuki Kanda,^3^ Hua Yang,^6^ Zeliang Qiu,^7^ Ruiyuan Hu,^8^ Xingao Li,^8^ Ammar Ahmed Khan,^9^ Yi Zhang,^2,3^ Jianxing Xia,^2,3*^ and Mohammad Khaja Nazeeruddin^3,10,11*^

1. School of Chemistry, Sun Yat-sen University, Guangzhou, Guangdong 510275, China

2. Institute of Molecular Plus, Tianjin University, Tianjin 300072, China

3. Institute of Chemical Sciences and Engineering, Ecole Polytechnique Federale de Lausanne (EPFL), CH-1951 Sion, Switzerland

4. Shenzhen Research Institute, Sun Yat-sen University, Shenzhen, Guangdong 518057, China

5. Instrumental Analysis & Research Center, Sun Yat-Sen University, Guangzhou, Guangdong 510275, China

6. Institute of High Energy Physics, Chinese Academy of Sciences (CAS), Beijing, 100049 China

7. College of Materials and Chemistry and Chemical Engineering, Chengdu University of Technology, Chengdu, Sichuan 610059, China

8. New Energy Technology Engineering Laboratory of Jiangsu Province, School of Science, Nanjing University of Posts and Telecommunications (NJUPT), Nanjing, Jiangsu 210023, China

9. Department of Physics, Lahore University of Management Sciences, Lahore, Punjab 54792, Pakistan

10. Department of Mechanical and Energy Engineering, College of Engineering, Imam Abdulrahman Bin Faisal University, Dammam, P.O. Box 1982, 34212, Saudi Arabia

11. School of integrated Circuits, Southeast University, Wuxi, Jiangsu 214061, China

**Corresponding Authors**

shaog@mail.sysu.edu.cn; xiaban@tju.edu.cn; mdkhaja.nazeeruddin@epfl.ch

**Table of Contents**

**1. General Information** 3

**2. Synthesis of WD03 and WD04** 7

**3. Thermal Properties** 20

**4. Photophysical Properties** 20

**5. Computational Results** 24

**6. Hole Mobility** 26

**7. X-ray Crystallography** 27

**8. XPS Results** 33

**9. Surface Morphologies of HTM Films** 33

**10.** **Fs-TA Spectra and the Fitting Parameters** 35

**11. Photovoltaic Parameters of PSCs** 36

**12. References** 39

# **1. General Information**

(1) Materials and Methods

Titanium(IV) isopropoxide, lithium bis(trifluoromethylsulfonyl)imide (Li-TFSI), tris[2-(1*H*-pyrazol-1-yl)-4-*tert*-butylpyridine]-cobalt(III) tris[bis(trifluoromethylsulfonyl)imide] (FK209), 4-*tert*-butylpyridine (*t*-BP), isopropanol (IPA), chlorobenzene, dimethyl sulfoxide (DMSO), and *N*,*N*-dimethylformamide (DMF) were purchased from Sigma-Aldrich. Mesoporous TiO_2_ (30-NRT), FAI, MAI, and MACl were purchased from Great Cell Solar. PbI_2_ was purchased from TCI. Spiro-OMeTAD was purchased from Merck. *N*,*N*-dimethylanilinium tetrakis(pentafluorophenyl)borate (DPB) was purchased from Aladdin. All chemicals were used as received without further purification.

All dry reactions were conducted with glassware flamed under vacuum and recharged with argon. Solvents were dried according to standard procedures. Thin-layer chromatography was performed with silica gel (GF254) pre-coated on glass substrates and visualized under UV light (254 or 365 nm). Column chromatography was carried out with silica gel (300–400 mesh).

(2) Fabrication of Perovskite Solar Cells (PSCs)

Chemically etched FTO glass (Nippon Sheet Glass) was cleaned with detergent solution, acetone, and ethanol successively. For the compact TiO_2_ (c-TiO_2_) layer, the c-TiO_2_ precursor solution was prepared as follows: 369 μL of titanium(IV) isopropoxide was added to 2.53 mL of ethanol, and 35 μL of 2 M HCl solution was added to 2.53 mL of ethanol in another vial simultaneously. The second solution was then added dropwise to the first solution with vigorous stirring. The c-TiO_2_ solution was spin-coated at 4000 rpm for 50 s, followed by heating at 150 °C for 15 min, and then the c-TiO_2_ films were gradually heated to 500 °C and annealed for 30 min. Mesoporous TiO_2_ (m-TiO_2_) paste was diluted with ethanol at a ratio of 1:10 (*w*/*w*) and spin-coated on the top of c-TiO_2_ substrates at 3000 rpm for 20 s. Finally, the coated substrates were heated at 500 °C for 20 min. Perovskite precursors (CsI: 10.39 mg; MAI: 12.72 mg; FAI: 247.68 mg; PbI_2_: 785.55 mg; MACl: 15.00 mg) were dissolved in 1 mL of DMSO/DMF (*V*/*V* = 1/4) and spin-coated on the substrates at 1000 rpm for 10 s and 5000 rpm for 30 s consecutively. During the spin-coating, 200 μL of chlorobenzene was dropped in 10 s at 5000 rpm. Perovskite films were annealed at 150 °C for 10 min. Phenethylammonium iodide (5 mg) was dissolved in IPA (1 mL) and spin-coated on the perovskite film at 3000 rpm for 20 s. The hole-transporting material (HTM) solution was prepared by dissolving spiro-OMeTAD (75 mg) and additives in 1 mL of chlorobenzene. As the additives for spiro-OMeTAD, 18 µL of Li-TFSI from the stock solution (520 mg in 1 mL of acetonitrile), 13 µL of FK209 (375 mg in 1 mL of acetonitrile), and 30 µL of *t*-BP were added. Dopant-free **WD03** and **WD04** solutions were formulated by dissolving 20 mg of each HTM in 1 mL of chlorobenzene. In the doped state, the dopant-to-HTM mass ratios of the new HTMs matched those of spiro-OMeTAD. The HTM layers were formed by spin-coating the solutions at 4000 rpm for 20 s. In the case of DPB modification, 5 mg of DPB was dissolved in 1 mL of IPA and then dynamically spin-coated on **WD03** film at 3000 rpm for 30 s.^[1]^ Finally, a 70-nm-thick Au electrode was deposited by thermal evaporation.

(3) Characterization

^1^H and ^13^C NMR spectra were recorded at 25 °C on a Bruker Avance neo 400 spectrometer (400 MHz for ^1^H and 100 MHz for ^13^C). Chemical shifts (*δ*) are given in parts per million (ppm) referenced to solvent residual signal. The following abbreviations are used to indicate multiplicity: s, singlet; d, doublet; t, triplet; dd, doublet of doublets; m, multiplet. High-resolution mass spectrometry (HRMS) spectra were acquired on a Bruker timsTOF mass spectrometer equipped with electrospray ionization (ESI) technique. Thermogravimetric analysis (TGA) was conducted on a NETZSCH TG 209 F1 Libra thermogravimetric analyzer from 33 to 900 °C at a heating rate of 10 °C/min under nitrogen atmosphere. Differential scanning calorimetry (DSC) measurements were performed on a NETZSCH DSC 214 Polymer differential scanning calorimeter at a heating rate of 10 °C/min under nitrogen atmosphere. UV–Vis absorption spectra were measured on a Biochrom Libra S60 spectrometer. Steady-state photoluminescence (PL) spectra were recorded on a SHIMADZU RF-5301PC spectrometer. Cyclic voltammetry measurements were conducted with a CH Instruments 660E electrochemical workstation utilizing a three-electrode electrochemical cell at a scan rate of 50 mV/s. Tetrabutylammonium hexafluorophosphate (0.1 M) was added as electrolyte, an Ag/AgNO_3_ (0.01 M in acetonitrile) electrode was used as reference, a platinum wire was used as working electrode, and a platinum sheet was used as counter electrode. Prior to measurement, the sample was degassed by bubbling with argon for 10 min. Grazing incidence wide-angle X-ray scattering data (GIWAXs) were obtained at 1W1A Diffuse X-ray Scattering Station, Beijing Synchrotron Radiation Facility (BSRF). The wavelength of the monochromatic light source was 1.54 Å, and the grazing incidence angle was 0.2°. The data were recorded by a DECTRIS EIGER 2M two-dimensional image plate detector. X-ray intensities were collected on a Bruker APEX-II CCD diffractometer with Ga *K*_α_ radiation (*λ* = 1.34138 Å) at 150 K using *φ* and *ω* scans method. PL mapping images were captured by a Renishaw inVia Qontor confocal Raman microscope at room temperature. Laser with excitation wavelength of 325 nm was used to excite the samples. The mapping area was 30 µm × 30 µm with a step of 1 µm in both *x* and *y* directions. X-ray photoelectron spectroscopy (XPS) spectra were recorded on a Thermo Fisher Scientific ESCALAB Xi+ X-ray photoelectron spectrometer under high vacuum with an Al *K*_α_ X-ray source (1486.6 eV) and an electron take-off angle of 45°. Core-level signals were calibrated by setting the binding energy of C *1s* at 284.8 eV. Scanning electron microscope (SEM) cross-sectional image of device and top-view images of HTM films spin-coated on perovskite were acquired on a GeminiSEM 500 field-emission scanning electron microscope. Atomic force microscopy (AFM) and Kelvin probe force microscopy (KPFM) images were obtained on a Bruker Dimension XR atomic force microscope. Femtosecond transient absorption (fs-TA) spectra were recorded by a femtosecond regeneratively amplified Ti:sapphire laser system (Coherent，Astrella-Tunable-F-1K) and an automated data acquisition system (Ultrafast Systems, Helios). PSCs were measured under AM 1.5 G one sun illumination (100 mW cm^−2^) with a solar simulator calibrated by a Si-reference cell certified by NREL. During the test, the cells were masked to standardize the active area (0.09 cm^2^) and minimize the influence of scattered light on small device. All measurements were characterized at room temperature and in air. The monochromatic incident photon-to-current efficiency (IPCE) spectra were recorded as a function of wavelength under a constant white light bias (~10 mW cm^−2^), which was supplied by an array of white light-emitting diodes. The excitation beam from a 300 W xenon lamp (ILC Technology) was focused through a Gemini 180 double monochromator (Jobin Yvon Ltd.) and chopped at ~2 Hz. The signals were recorded using a SR830 DSP lock-in amplifier (Stanford Research Systems). Water contact angles were measured using a Krüss DSA 100 drop shape analyzer.

(4) Density Functional Theory (DFT) Calculations

DFT calculations were implemented by Gaussian 09W using B3LYP exchange-correlation functional and 6-31G basis set.^[2]^ The results were visualized by GaussView.^[3]^ The geometry optimization of HTMs on the perovskite was performed by Vienna *Ab*-*inito* Simulation Package (VASP) using Perdew–Burke–Ernzerhof exchange-correlation potential and projector augmented wave method.^[4–6]^ The optimizations of the lattice constants and the atomic coordinates were made by the minimization of the total energy with a 2 × 2 × 2 k-point grid. All the atoms were allowed to relax until all internal atomic forces fell within 0.005 eV Å^−1^. The plane-wave cutoff energy was set as 600 eV.

# **2. Synthesis of WD03 and WD04**

**Scheme S1.** Synthetic routes of **WD03** and **WD04**.

(1) Synthesis of **1**

4-Bromothioanisole (2.4303 g, 11.966 mmol, 1.00 equiv.), tris(dibenzylideneacetone) dipalladium (0.5617 g, 0.613 mmol, 0.05 equiv.), sodium *tert*-butoxide (2.4751 g, 25.755 mmol, 2.15 equiv.), 4-(methylthio)aniline (1.8955 g, 13.615 mmol, 1.14 equiv.), toluene (30 mL), and tri-*tert*-butylphosphine solution (200 mg/mL in toluene, 4.8 mL, 4.745 mmol, 0.40 equiv.) were added into a two-necked flask. The mixture was heated to 90 °C and stirred for 23 h in an argon atmosphere. The solvent was removed by vacuum rotary evaporation, and the residue was subjected to column chromatography (eluent: petroleum ether/ethyl acetate = 20/1, *V*/*V*) to afford a brown solid (2.6740 g, 85.49%). ^1^H NMR (400 MHz, DMSO-*d*_6_): *δ* (ppm) = 8.08 (s, 1H), 7.16 (d, *J* = 8.0 Hz, 4H), 6.98 (d, *J* = 8.0 Hz, 4H), 2.36 (s, 6H), coinciding with the reported data.^[7]^

**Figure** **S1.** ^1^H NMR spectrum of **1** (400 MHz, DMSO-*d*_6_).

(2) Synthesis of **2**

5-Bromo-2-thiophenecarboxylic acid (0.5251 g, 2.536 mmol, 1.00 equiv.) and thionyl chloride (2 mL, 27.572 mmol, 10.87 equiv.) were added into a one-necked flask. The mixture was heated to 100 °C and refluxed with stirring for 4 h in an argon atmosphere. Excessive thionyl chloride was removed by vacuum rotary evaporation. 2-Bromothiophene (0.8300 g, 5.091 mmol, 2.01 equiv.), dichloromethane (4 mL), and aluminum chloride (0.7385 g, 5.538 mmol, 2.18 equiv.) were added to the residue. The mixture was stirred for 18 h in an argon atmosphere at room temperature. The reaction was quenched by water and the resulting mixture was extracted with dichloromethane. The extract was dried by anhydrous magnesium sulfate, filtered, and evaporated under a reduced pressure. The crude product was purified by column chromatography (eluent: petroleum ether/ethyl acetate = 50/1, *V*/*V*) to provide an orange solid (0.5497 g, 61.57%). ^1^H NMR (400 MHz, CDCl_3_): *δ* (ppm) = 7.60 (d, *J* = 4.0 Hz, 2H), 7.16 (d, *J* = 4.0 Hz, 2H), coinciding with the reported data.^[8]^

**Figure** **S2.** ^1^H NMR spectrum of **2** (400 MHz, CDCl_3_).

(3) Synthesis of **3**

Under an argon atmosphere, a solution of **2** (1.0137 g, 2.879 mmol, 1.00 equiv.) in methanol (20 mL) was cooled down in an ice bath. Next, sodium borohydride (1.0975 g, 29.011 mmol, 10.08 equiv.) was added and the mixture was stirred for 13 h at room temperature. The reaction was quenched by water and the resulting mixture was extracted by ethyl acetate. The extract was dried by anhydrous magnesium sulfate, filtered, and concentrated to dryness under a reduced pressure. The crude product was purified by column chromatography (eluent: petroleum ether/ethyl acetate = 10/1, *V*/*V*) to give a yellow oil (1.0100 g, 99.08%). ^1^H NMR (400 MHz, CDCl_3_): *δ* (ppm) = 6.93 (d, *J* = 4.0 Hz, 2H), 6.77 (d, *J* = 4.0, 1.0 Hz, 2H), 6.10 (s, 1H), 2.66 (s, 1H). ^13^C NMR (100 MHz, CDCl_3_): *δ* (ppm) = 144.82, 129.61, 126.92, 113.92, 72.84.

**Figure** **S3.** ^1^H NMR spectrum of **3** (400 MHz, CDCl_3_).

**Figure** **S4.** ^13^C NMR spectrum of **3** (100 MHz, CDCl_3_).

(4) Synthesis of **4**

Compound **3** (1.2111 g, 3.421 mmol, 1.00 equiv.), triethyl phosphite (1.8 mL, 10.356 mmol, 3.03 equiv.), and zinc bromide (1.6707 g, 7.419 mmol, 2.17 equiv.) were added into a one-necked flask. The mixture was heated to 45 °C and stirred for 22 h in an argon atmosphere. After that, the crude product was subjected to column chromatography (eluent: petroleum ether/ethyl acetate = 5/1, *V/V*) to yield a yellow oil (1.2424 g, 76.59%). ^1^H NMR (400 MHz, CDCl_3_): *δ* (ppm) = 6.93–6.91 (m, 4H), 4.74 (d, *J* = 25.6 Hz, 1H), 4.14–3.94 (m, 4H), 1.23 (t, *J* = 6.8 Hz, 6H). ^13^C NMR (100 MHz, CDCl_3_): *δ* (ppm) = 139.36 (d, *J* = 5.9 Hz), 129.89 (d, *J* = 2.9 Hz), 127.95 (d, *J* = 8.1 Hz). 112.22 (d, *J* = 3.6 Hz), 63.72 (d, *J* = 7.3 Hz), 41.92 (d, *J* = 144.3 Hz), 16.45 (d, *J* = 5.8 Hz).

**Figure** **S5.** ^1^H NMR spectrum of **4** (400 MHz, CDCl_3_).

**Figure S6.** ^13^C NMR spectrum of **4** (100 MHz, CDCl_3_).

(5) Synthesis of **5**

A mixture of **4** (1.5005 g, 3.165 mmol, 1.00 equiv.), 5-bromo-2-thiophenecarboxaldehyde (1.1708 g, 6.129 mmol, 1.94 equiv.), and tetrahydrofuran (20 mL) in a two-necked flask was cooled down in an ice bath. After sodium hydride (60% dispersion in mineral oil, 0.5611 g, 14.028 mmol, 4.43 equiv.) was added, the mixture was stirred for 23 h in an argon atmosphere at room temperature. The solvent was evaporated by vacuum rotary evaporation, and the residue was subjected to column chromatography (eluent: petroleum ether) to provide a yellow solid (1.3142 g, 81.24%). ^1^H NMR (400 MHz, CDCl_3_): *δ* (ppm) = 7.14 (d, *J* = 3.6 Hz, 1H), 7.07 (s, 1H), 6.91 (dd, *J* = 4.0, 2.0 Hz, 2H), 6.82 (t, *J* = 3.6 Hz, 2H), 6.58 (d, *J* = 4.0 Hz, 1H). ^13^C NMR (100 MHz, CDCl_3_): *δ* (ppm) = 147.20, 141.11, 138.50, 130.87, 130.80, 130.54, 130.18, 129.42, 126.21, 124.70, 123.29, 115.94, 114.96, 112.31.

**Figure** **S7.** ^1^H NMR spectrum of **5** (400 MHz, CDCl_3_).

**Figure** **S8.** ^13^C NMR spectrum of **5** (100 MHz, CDCl_3_).

(6) Synthesis of **6**

Compound **6** was synthesized according to previous work.^[9]^

(7) Synthesis of **WD03**

Compound **6** (0.6107 g, 1.239 mmol, 1.00 equiv.), compound **1** (1.0188 g, 3.897 mmol, 3.15 equiv.), tris(dibenzylideneacetone) dipalladium (0.1135 g, 0.124 mmol, 0.10 equiv.), sodium *tert*-butoxide (0.5968 g, 6.210 mmol, 5.01 equiv.), toluene (50 mL), and tri-*tert*-butylphosphine solution (100 mg/mL in toluene, 2.0 mL, 0.989 mmol, 0.80 equiv.) were added into a two-necked flask. The mixture was heated to 90 °C and stirred for 12 h in an argon atmosphere. The solvent was removed by vacuum rotary evaporation, and the residue was subjected to column chromatography (eluent: petroleum ether/dichloromethane = 2/1, *V*/*V*) to yield a yellow solid (0.8664 g, 67.60%). ^1^H NMR (400 MHz, CDCl_3_): *δ* (ppm) = 7.22 (d, *J* = 8.8 Hz, 2H), 7.19–7.16 (m, 12H), 7.10 (d, *J* = 8.8 Hz, 2H), 7.05–6.97 (m, 16H), 6.94 (d, *J* = 8.8 Hz, 2H), 6.84–6.82 (m, 3H), 2.47 (m, 18H). ^13^C NMR (100 MHz, CDCl_3_): *δ* (ppm) = 146.78, 146.68, 145.87, 145.21, 145.09, 140.20, 137.57, 134.68, 132.21, 132.12, 132.06, 131.36, 130.42, 128.67, 128.63, 128.59, 128.24, 126.22, 125.07, 125.01, 123.46, 122.93, 122.48, 16.97, 16.93. HRMS (ESI) *m*/*z*: [M]^+^ Calcd for C_62_H_55_N_3_S_6_, 1033.2715; Found, 1033.2704.

**Figure** **S9.** ^1^H NMR spectrum of **WD03** (400 MHz, CDCl_3_).

**Figure** **S10.** ^13^C NMR spectrum of **WD03** (100 MHz, CDCl_3_).


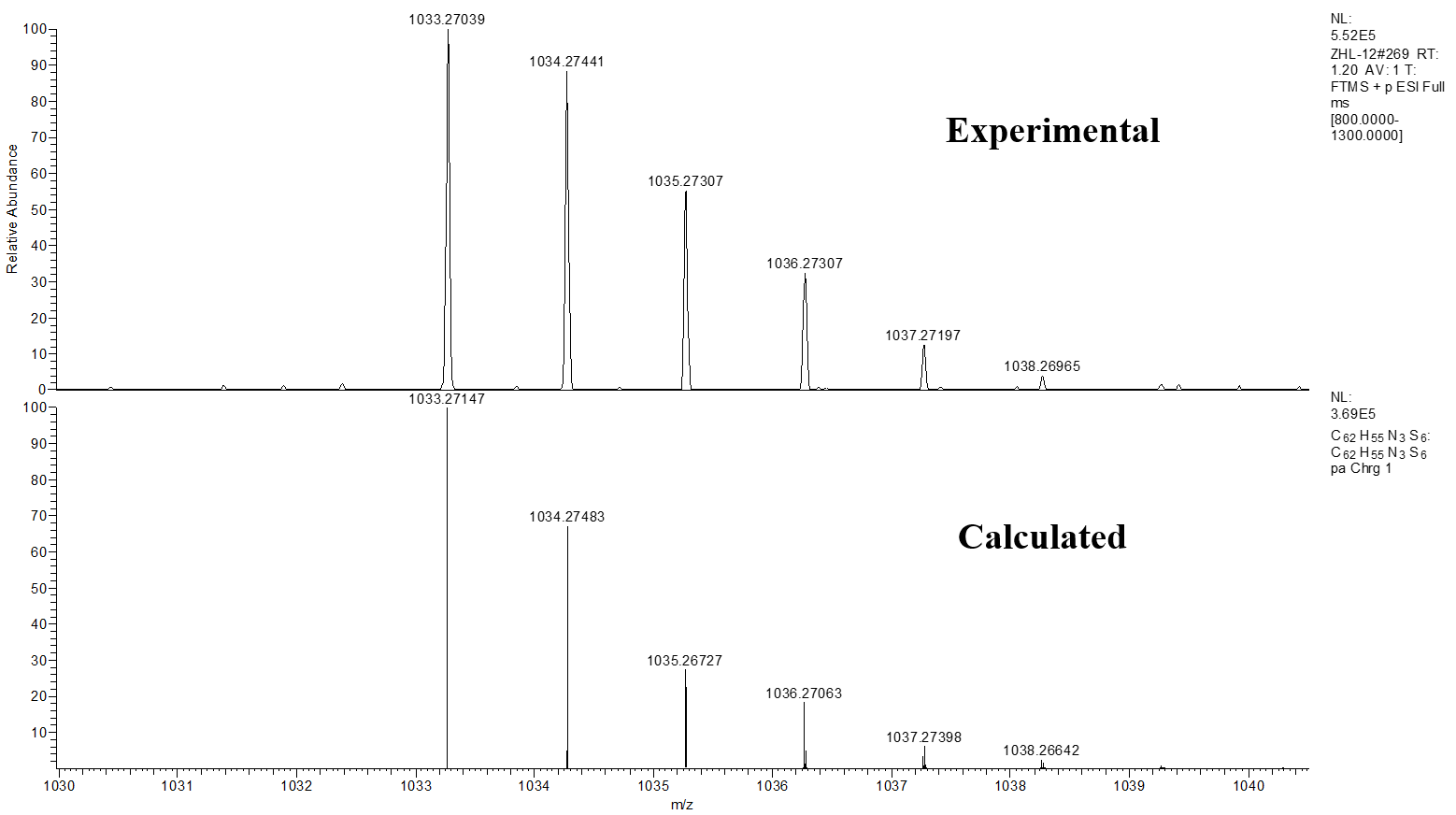


**Figure** **S11.** HRMS spectrum of **WD03**.

(8) Synthesis of **WD04**

Compound **5** (0.2146 g, 0.420 mmol, 1.00 equiv.), compound **1** (0.4060 g, 1.553 mmol, 3.70 equiv.), tris(dibenzylideneacetone) dipalladium (0.0652 g, 0.071 mmol, 0.17 equiv.), tri-*tert*-butylphosphonium tetrafluoroborate (0.1078 g, 0.372 mmol, 0.89 equiv.), cesium carbonate (0.1344 g, 0.412 mmol, 0.98 equiv.), sodium *tert*-butoxide (0.2699 g, 2.809 mmol, 6.69 equiv.), and toluene (10 mL) were added into a two-necked flask. The mixture was heated to 90 °C and stirred for 23 h in an argon atmosphere. The solvent was removed by vacuum rotary evaporation, and the residue was subjected to column chromatography (eluent: petroleum ether/dichloromethane = 3/1, *V*/*V*) to produce an orange solid (0.1905 g, 43.09%). ^1^H NMR (400 MHz, CDCl_3_): *δ* (ppm) = 7.20–7.17 (m, 8H), 7.10–6.99 (m, 16H), 6.94 (s, 1H), 6.75 (d, *J* = 4.0 Hz, 2H), 6.55 (dd, *J* = 11.2, 4.0 Hz, 2H), 6.46 (d, *J* = 4.0 Hz, 1H), 6.39 (d, *J* = 4.0 Hz 1H), 2.47–2.43 (m, 18H). ^13^C NMR (100 MHz, CDCl_3_): *δ* (ppm) = 153.30, 152.93, 150.25, 145.44, 145.19, 145.10, 140.05, 133.94, 132.78, 132.74, 132.34, 130.91, 128.79, 128.60, 128.52, 128.48, 128.34, 124.67, 124.58, 123.59, 123.41, 122.39, 120.21, 118.78, 16.99, 16.92, 16.83. HRMS (ESI) *m*/*z*: [M]^+^ Calcd for C_56_H_49_N_3_S_9_, 1051.1407; Found, 1051.1395.

**Figure** **S12.** ^1^H NMR spectrum of **WD04** (400 MHz, CDCl_3_).

**Figure** **S13.** ^13^C NMR spectrum of **WD04** (100 MHz, CDCl_3_).


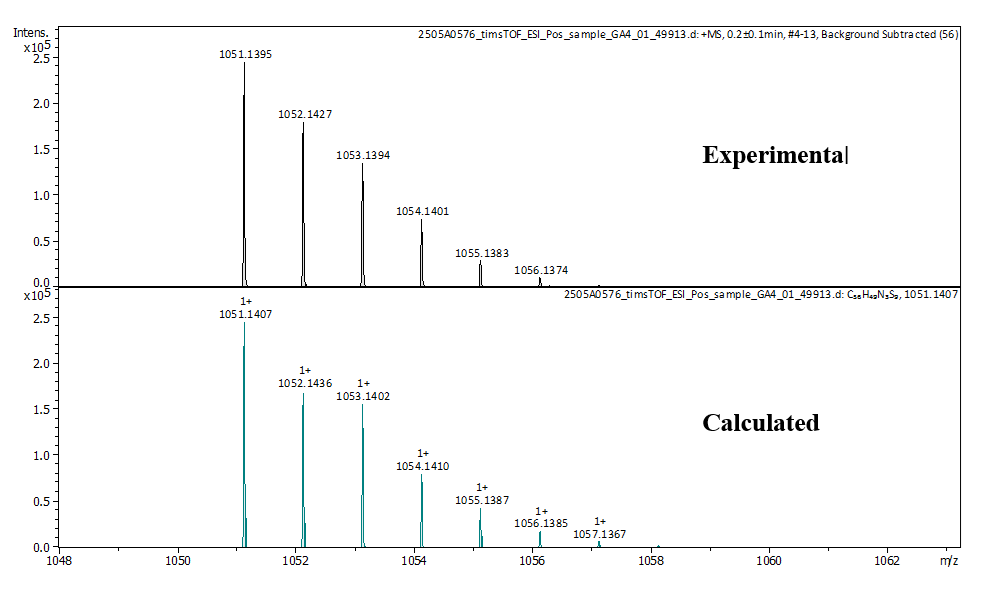


**Figure** **S14.** HRMS spectrum of **WD04**.

# **3. Thermal Properties**







**Figure S15.** DSC of **WD03** and **WD04**.

# **4. Photophysical Properties**

**Table S1.** Photophysical properties of HTM solutions and films

| HTM | *λ*_abs_ (solution, nm) | *λ*_abs_ (film, nm) |
| --- | --- | --- |
| **WD03** | 256, 326 (max), 395 | 252, 330 (max), 399 |
| **WD04** | 264, 321 (max), 450 | 260, 324 (max), 459 |
| spiro-OMeTAD | 308, 385 (max) | 308, 386 (max) |










**Figure S16.** UV–Vis absorption spectra of **WD03**, **WD04**, and spiro-OMeTAD in CH_2_Cl_2_ (1.0 × 10^−5^ M) and in films.





**Figure S17.** PL spectra of **WD03** in CH_2_Cl_2_ (1.0 × 10^−5^ M; *λ*_ex_ = 333 nm, *λ*_em_ = 501 nm; slit: 3.0 nm, 3.0 nm).





**Figure S18.** PL spectra of **WD04** in CH_2_Cl_2_ (1.0 × 10^−5^ M; *λ*_ex_ = 325 nm, *λ*_em_ = 525 nm; slit: 5.0 nm, 10.0 nm).





**Figure S19.** PL spectra of spiro-OMeTAD in CH_2_Cl_2_ (1.0 × 10^−5^ M; *λ*_ex_ = 392 nm, *λ*_em_ = 422 nm; slit: 1.5 nm, 1.5 nm).










**Figure S20.** Normalized UV–Vis absorption and emission spectra of **WD03**, **WD04**, and spiro-OMeTAD in CH_2_Cl_2_ (1.0 × 10^−5^ M).

# **5. Computational Results**

**Table S2**. Optimized molecular configurations and frontier orbitals of HTMs obtained from DFT calculations

| HTM | optimized molecular  configuration | HOMO | LUMO |
| --- | --- | --- | --- |
| **WD03** | 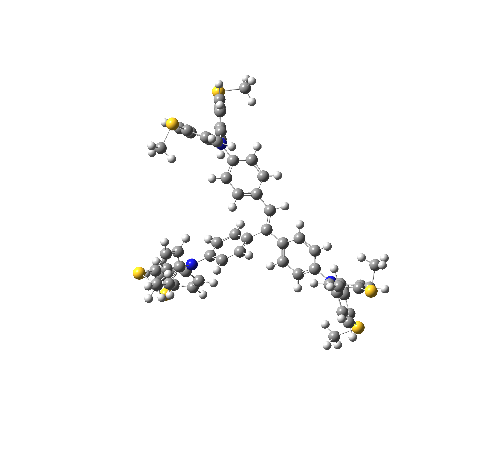 | 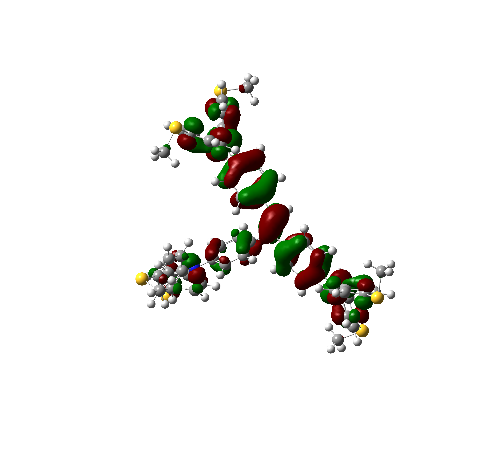 | 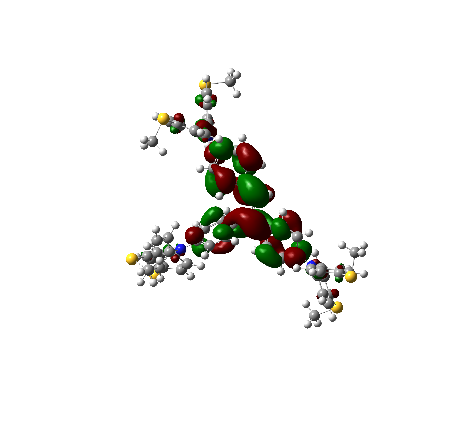 |
| **WD04** | 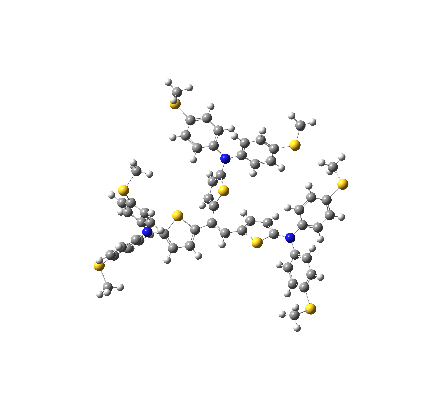 | 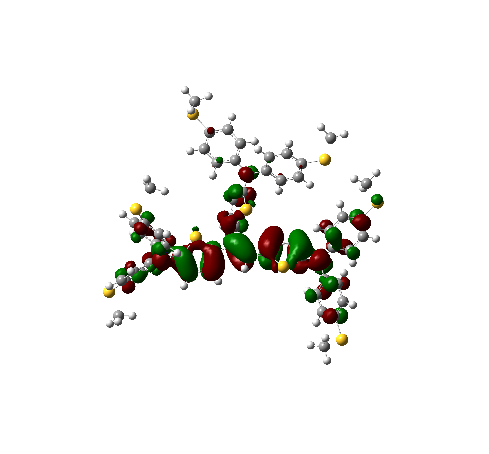 | 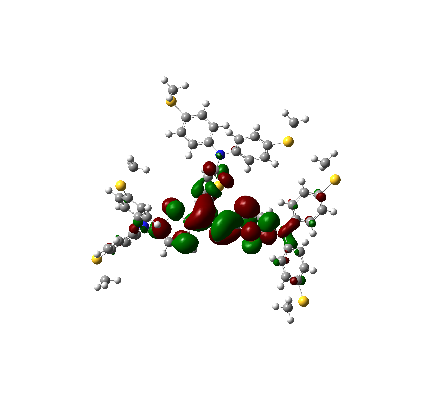 |
| spiro-OMeTAD | 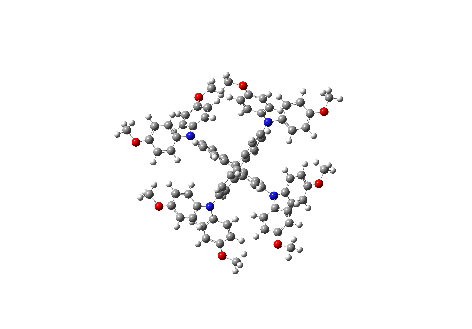 | 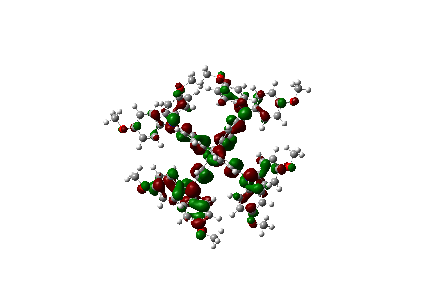 | 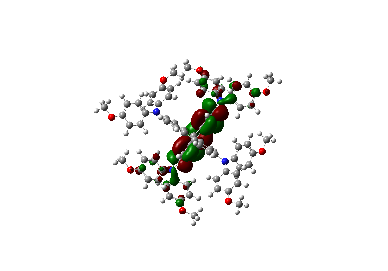 |

**Table S3.** Calculated energy levels of HTMs

| HTM | HOMO (eV) | LUMO (eV) | *E*_gap_ (eV) |
| --- | --- | --- | --- |
| **WD03** | −5.01 | −1.63 | −3.38 |
| **WD04** | −4.86 | −2.07 | −2.79 |
| spiro-OMeTAD | −4.33 | −0.71 | −3.62 |

**Table S4.** Electrostatic surface potential (ESP) maps and dipole moment of HTMs

| HTM | dipole moment (D) | | ESP map |  |
| --- | --- | --- | --- | --- |
| **WD03** | 1.99 | 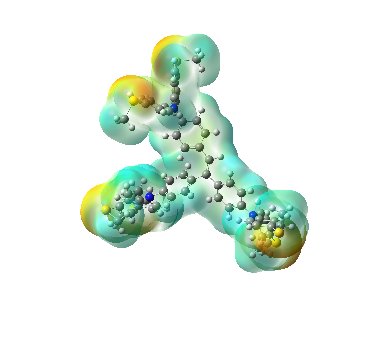 | | 0.042  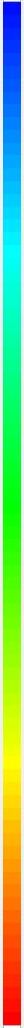  −0.042 |
| **WD04** | 2.50 | **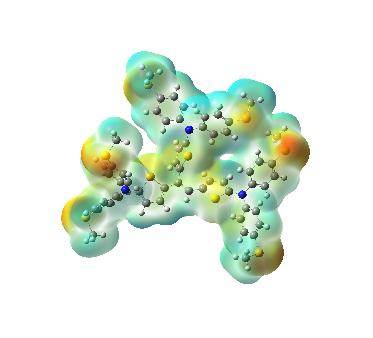** | |  |
| spiro-OMeTAD | 6.19 | 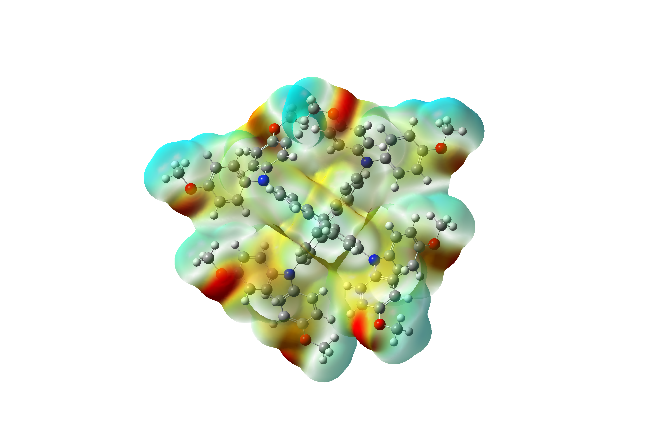 | |  |

# **6. Hole Mobility**

Hole mobility of HTMs was measured using space-charge-limited current (SCLC) method. Single carrier hole-only devices (FTO/PEDOT:PSS/HTM/Au) were fabricated to conduct the measurements. FTO glass was cleaned sequentially with detergent, deionized water, acetone, and ethanol in ultrasonic bath for 15 min, and finally cleaned with ultraviolet ozone for 20 min. PEDOT:PSS (40 nm) was spin-coated at 3000 rpm for 30 s and annealed at 150 °C for 20 min. The HTM layers were formed by spin-coating the chlorobenzene solutions (20 mg/mL) at 2000 rpm for 30 s. An Au layer (70 nm) was thermally evaporated under high vacuum on the top of the HTM layer as the cathode. The active area of device was 0.16 cm^2^, which was defined by the overlapping area of the FTO and Au electrodes. The thicknesses of HTM films were measured by a Surfcorder ET150 stylus profilometer (Kosaka Laboratory Ltd.). The current density–voltage (*J*–*V*) characteristics were recorded on a CHI660E electrochemical workstation. The SCLC characteristics in trap-free regime can be described by Mott–Gurney law:

Where *J* is the current density, *ε*_r_ is the relative permittivity and assumed approximately to be 3 for organic semiconductor, *ε*_0_ is the permittivity of the free space (8.85 × 10^−12^ F m^−1^), *μ* is the hole mobility, *V* is the effective voltage (*V* = *V*_appl_ − *V*_bis_ − *V*_s_, where *V*_appl_ is the applied voltage, *V*_bis_ is the built-in voltage, and *V*_s_ is the voltage drop resulting from the series resistance), and *d* is the thickness of HTM film.










**Figure S21.** *J*^1/2^–*V* characteristics of hole-only devices containing **WD03**, **WD04**, and spiro-OMeTAD, respectively.

# **7. X-ray Crystallography**

A single crystal of **WD03** was selected from solution and coated with Paratone oil. Then it was mounted on a glass fiber, fixed onto a stainless steel holder, and rapidly loaded into the diffractometer. The structure was solved by direct methods and refined by full-matrix least squares technique against *F*^2^ using SHELXT and SHELXL, respectively. Tertiary, secondary, and aromatic hydrogen atoms were refined utilizing riding model with isotropic thermal parameters: *U*_iso_ (H) = 1.2 *U*_eq_ (CH). Idealized –CH_3_ was refined as a rotating group with isotropic thermal parameters: *U*_iso_ (H) = 1.5 *U*_eq_ (CH_3_). Crystal and refinement parameters are listed in Table S5.


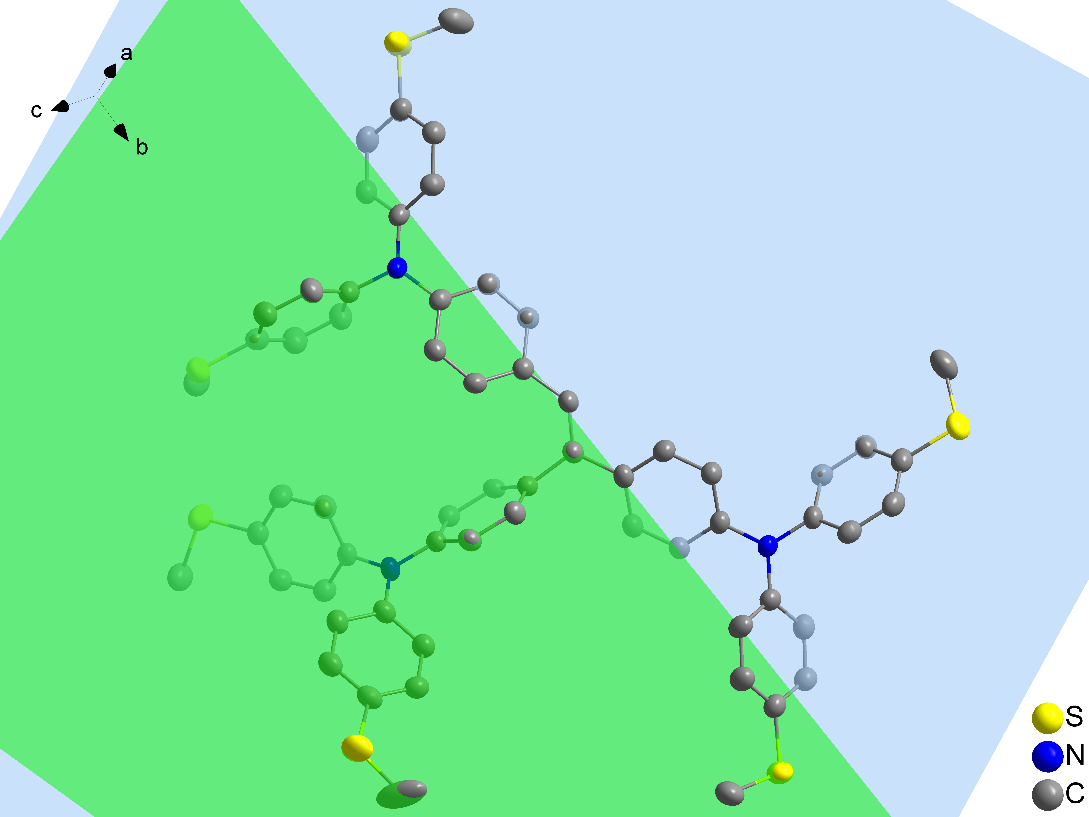


20.05°

Mean plane: C9, C8, C7, C15, C4 (blue)

Mean plane: C9, C10, C11, C12, C13, C14 (green)

C4

C13

C14

C12

C11

C10

C15

C7

C8

C9

**Figure S22.** Dihedral angle (20.05°) between double bond and benzene ring (H atoms were omitted for clarity.).


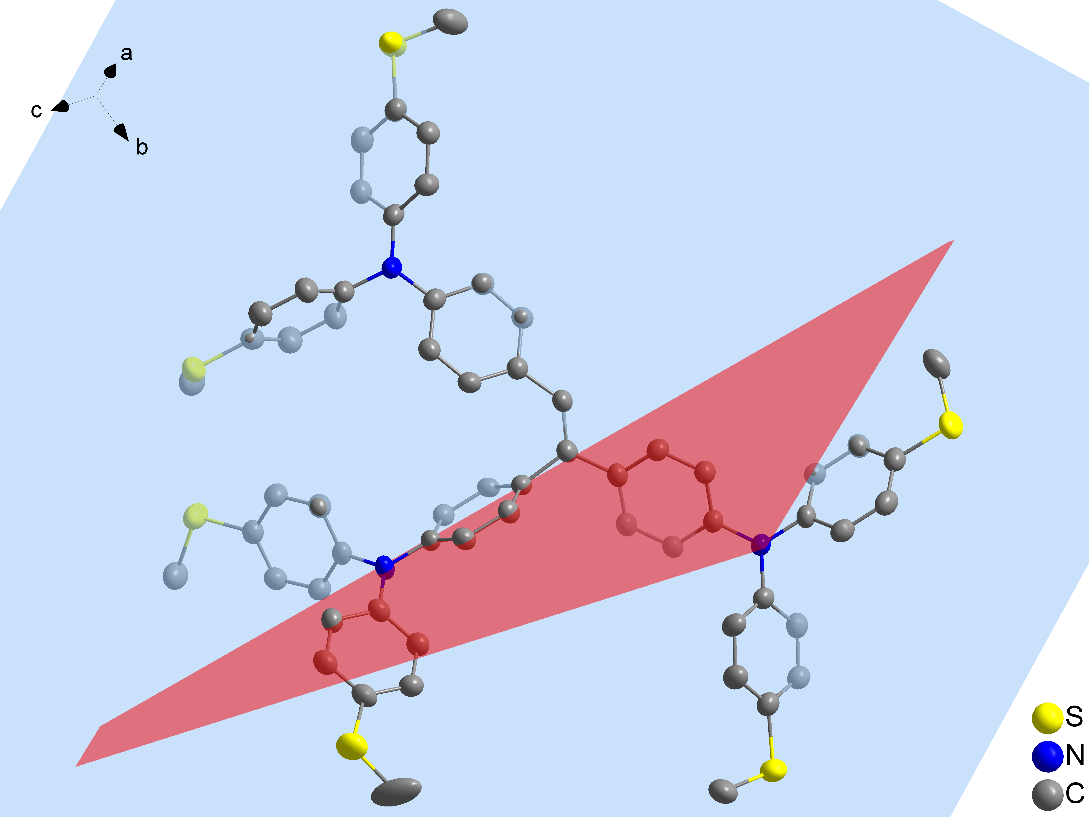


66.84°

Mean plane: C9, C8, C7, C15, C4 (blue)

Mean plane: C15, C16, C17, C18, C19, C20 (red)

C19

C20

C18

C17

C16

C15

C4

C7

C9

C8

**Figure S23.** Dihedral angle (66.84°) between double bond and benzene ring (H atoms were omitted for clarity.).


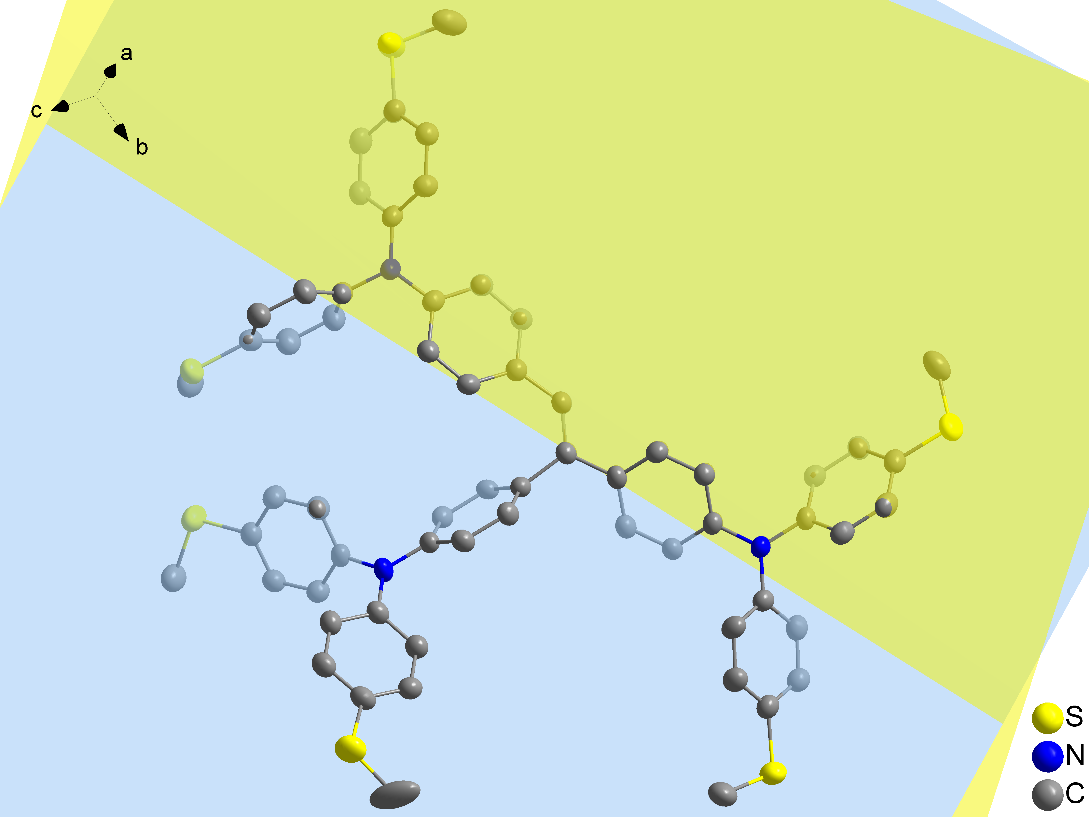


Mean plane: C9, C8, C7, C15, C4 (blue)

Mean plane: C1, C2, C3, C4, C5, C6 (yellow)

C5

C6

C1

C2

C3

C4

C15

C7

C8

C9

27.40°

**Figure S24.** Dihedral angle (27.40°) between double bond and benzene ring (H atoms were omitted for clarity.).


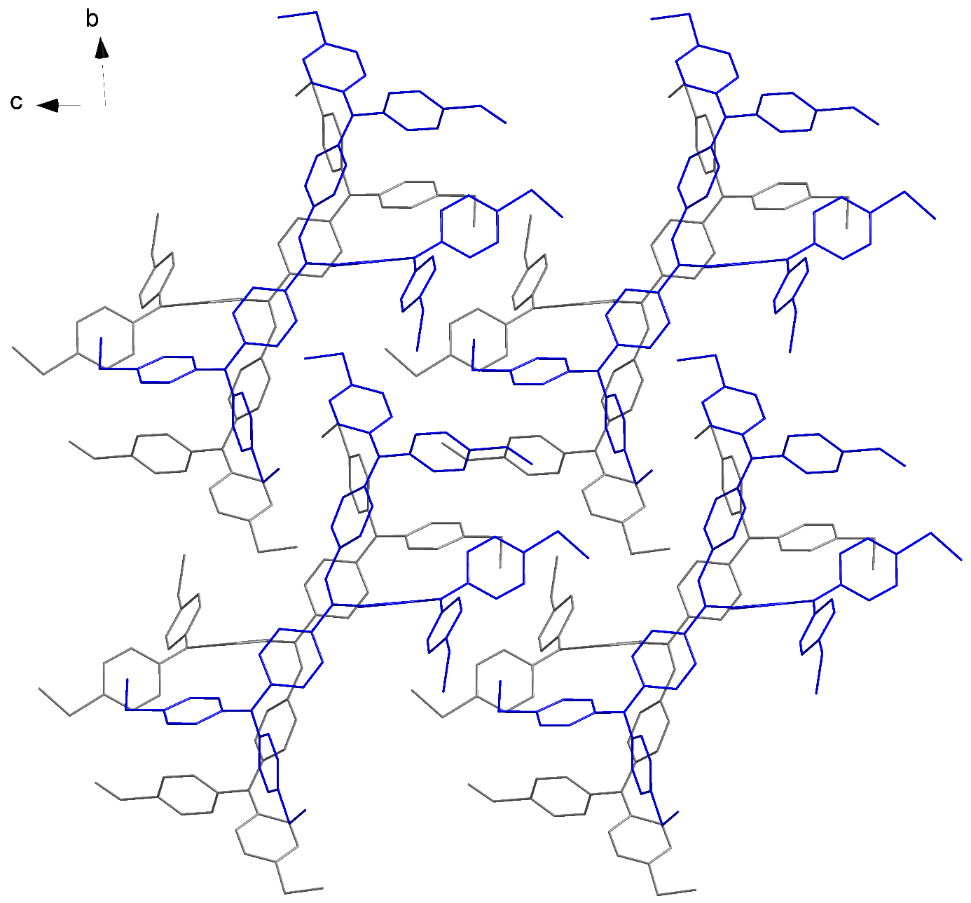


**Figure S25.** Molecular packing of **WD03** along the *a* axis (H atoms were omitted for clarity.).


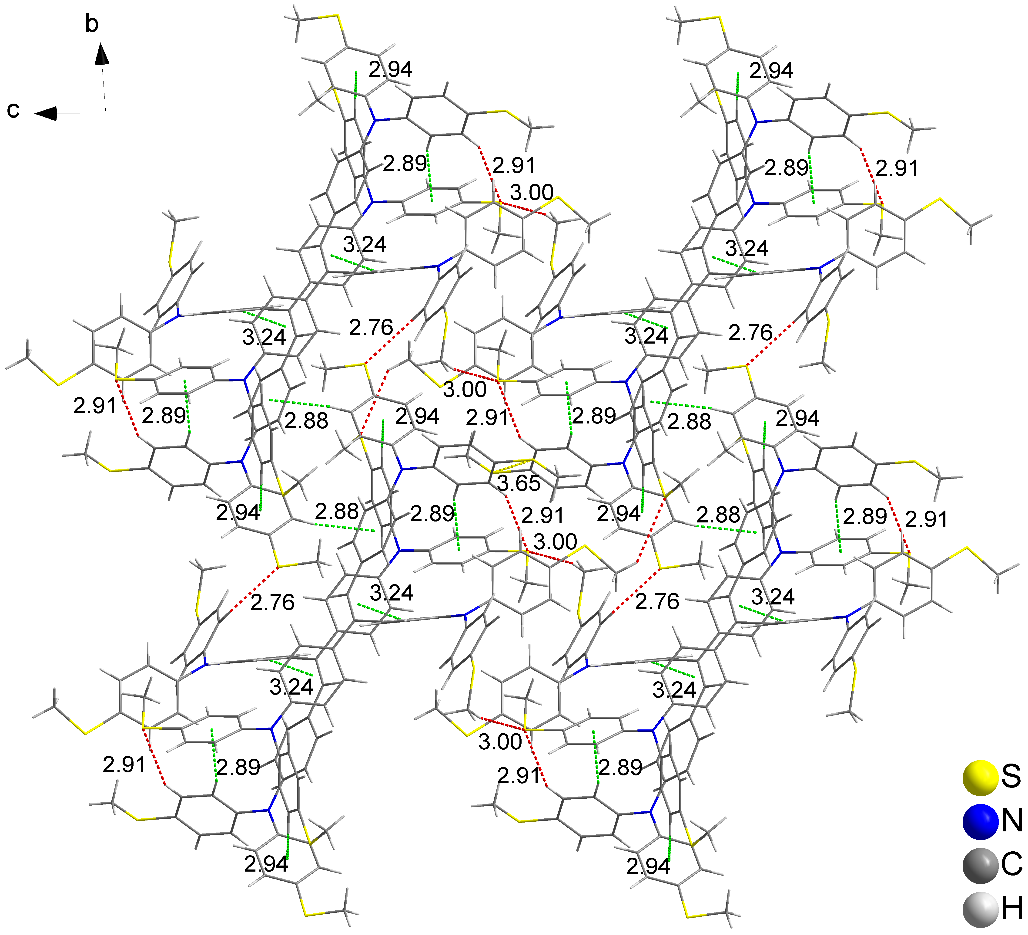


**Figure S26.** S‧‧‧S (yellow dashed line), CH‧‧‧S (red dashed lines), and CH‧‧‧*π* (green dashed lines) interactions in the **WD03** crystal (unit: Å).


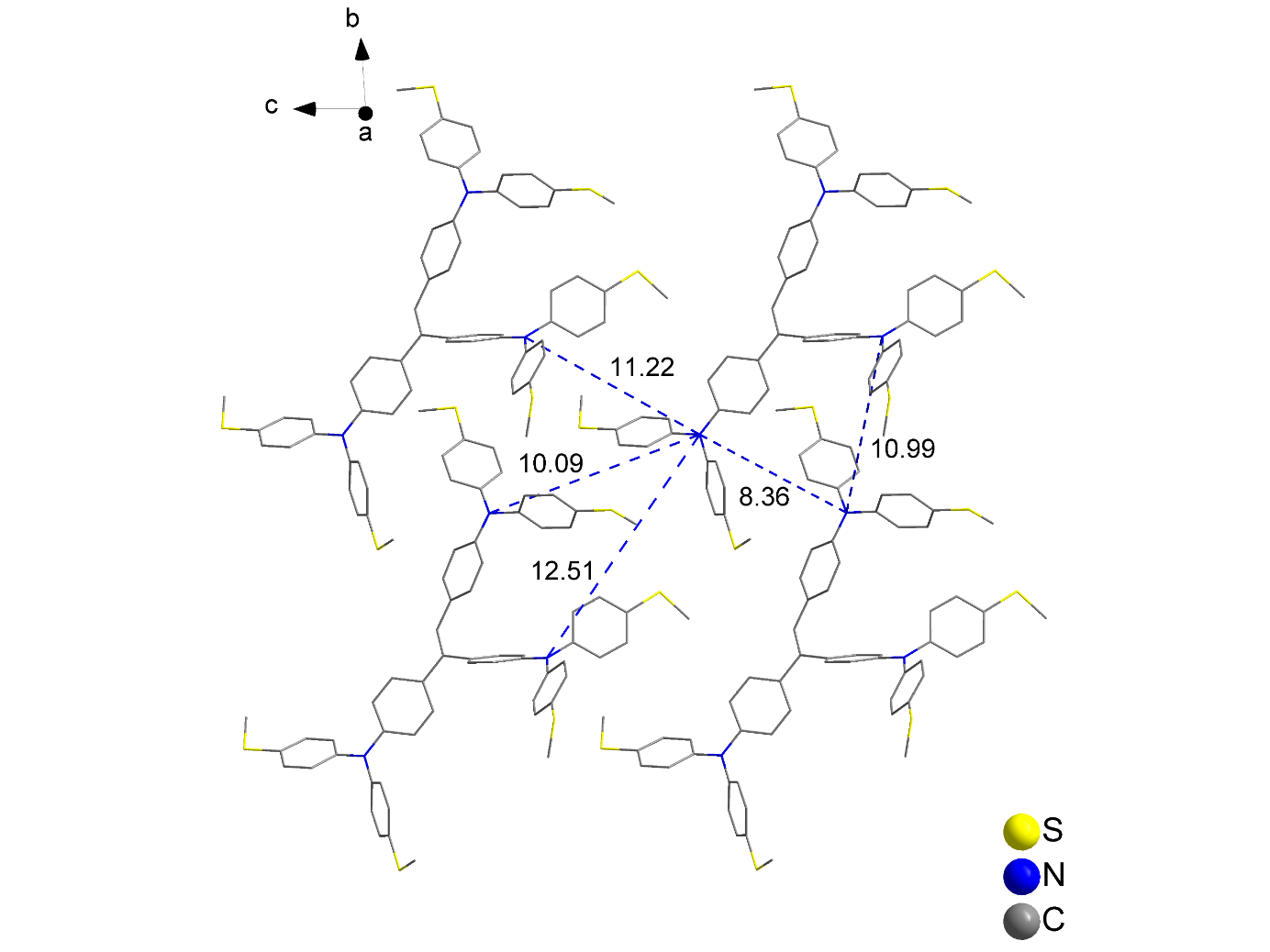


**Figure S27.** N–N distances within a layer of **WD03** molecules (dashed blue line; unit: Å; H atoms were omitted for clarity.).

**Table S5.** Crystallographic parameters of **WD03**

| substance | **WD03** |
| --- | --- |
| formula | C_62_H_55_N_3_S_6_ |
| *M*_r_ | 1034.45 |
| crystal color, habit | yellow, needle |
| crystal system, space group | triclinic, *P*$\bar{1}$ |
| radiation type, wavelength (Å) | Ga *K*_α_, 1.34138 |
| temperature (K) | 150.00 |
| crystal size (mm) | 0.021 × 0.006 × 0.005 |
| *a* (Å) | 11.3457(15) |
| *b* (Å) | 14.877(2) |
| *c* (Å) | 16.291(3) |
| *α* (°) | 83.693(6) |
| *β* (°) | 79.038(7) |
| *γ* (°) | 80.614(8) |
| *V* (Å^3^) | 2654.6(7) |
| *ρ* (calc., g cm^−3^) | 1.294 |
| *Z* | 2 |
| *μ* (mm^−1^) | 1.783 |
| N° of rflcn/unique | 42780/11574 |
| *θ* range (°) | 2.411–59.489 |
| compl. to *θ*_max_ (%) | 98.4 |
| *R*_1_/*wR*_2_ [*I* > 2*σ* (*I*)] | 0.0689/0.1722 |
| *R*_1_/*wR*_2_ (all data) | 0.1330/0.2063 |
| *Goof* | 1.030 |
| *R*_int_ | 0.0793 |
| largest diff. peak and hole (eÅ^−3^) | 0.847 and −0.997 |

# **
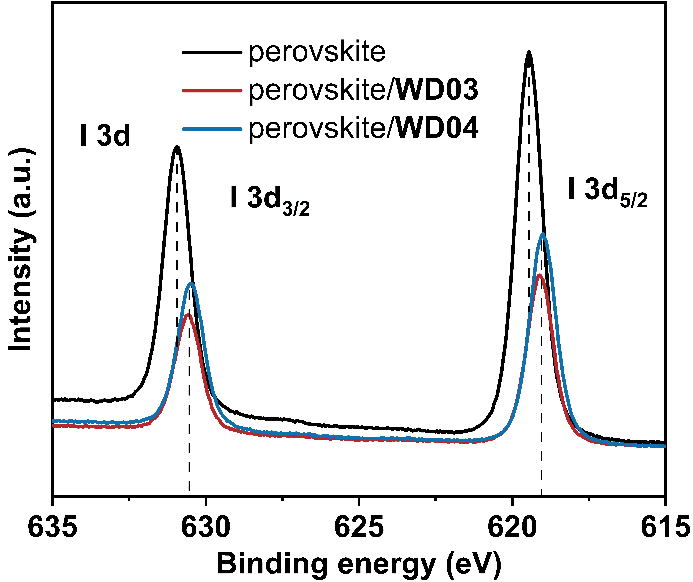
8. XPS Results**

**Figure S28.** XPS spectra of perovskite film and perovskite films spin-coated with **WD03** and **WD04**, respectively.

# **9. Surface Morphologies of HTM Films**

|  | **Low magnification** | **High magnification** |
| --- | --- | --- |
| (a) | 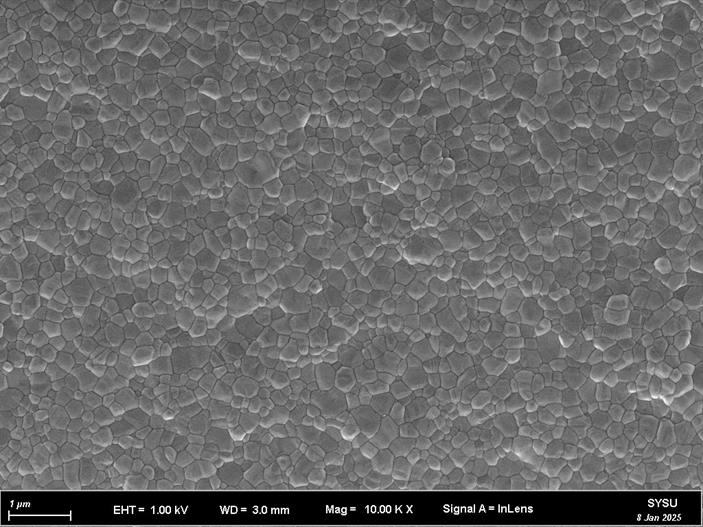 | 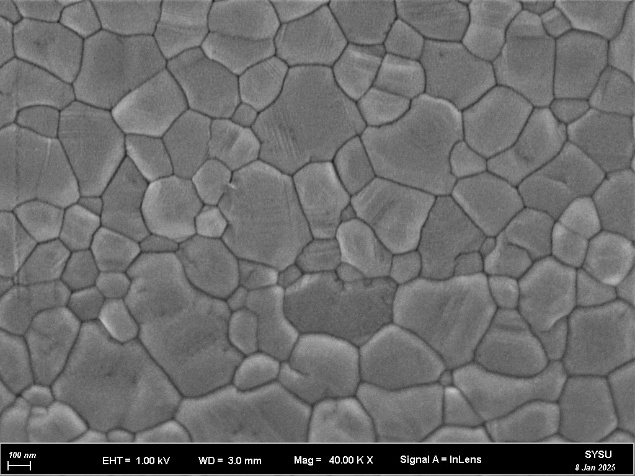 |
| (b) | 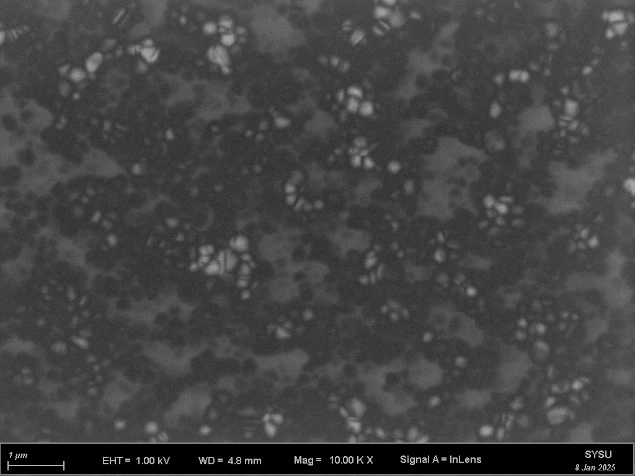 | 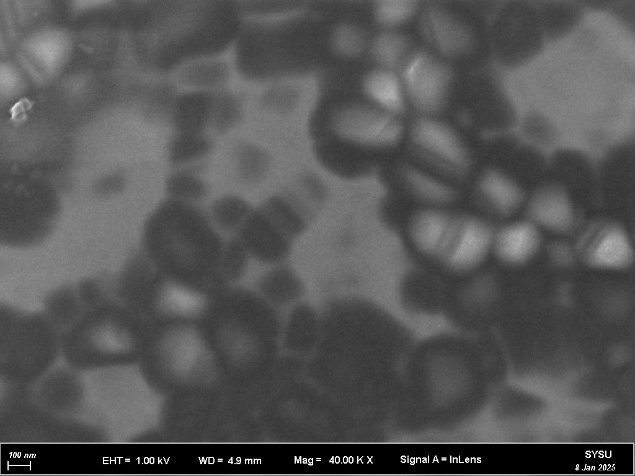 |
| (c) | 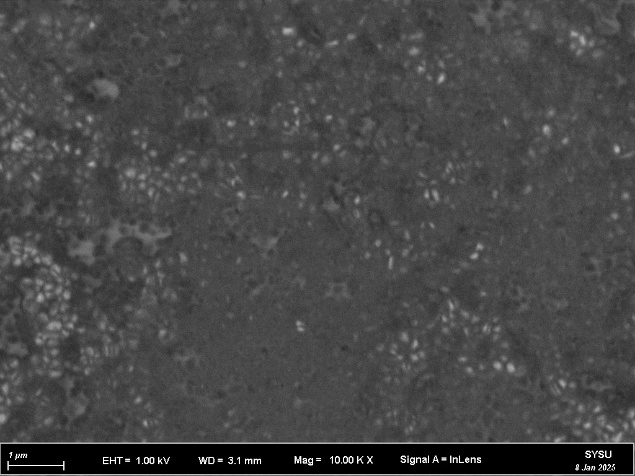 | 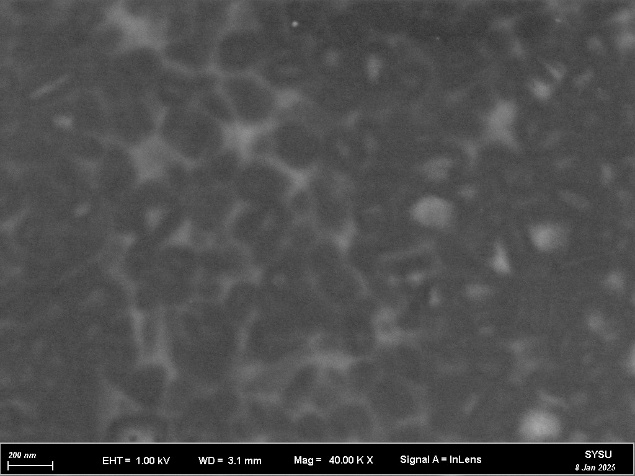 |
| (d) | 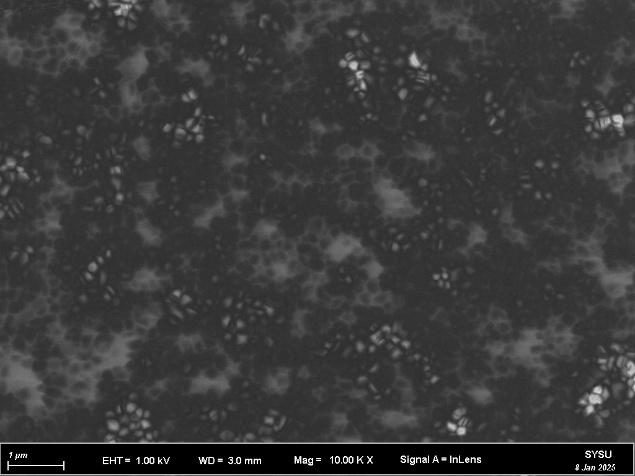 | 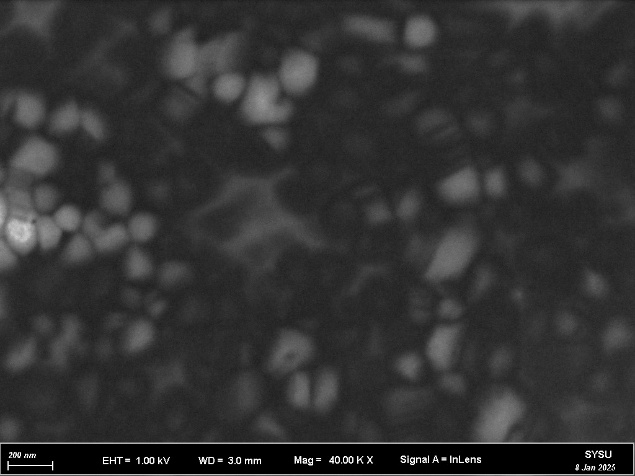 |

**Figure S29.** SEM top-view images of HTM films spin-coated on perovskite: (a) perovskite, (b) perovskite/**WD03**, (c) perovskite/**WD04**, and (d) perovskite/spiro-OMeTAD.


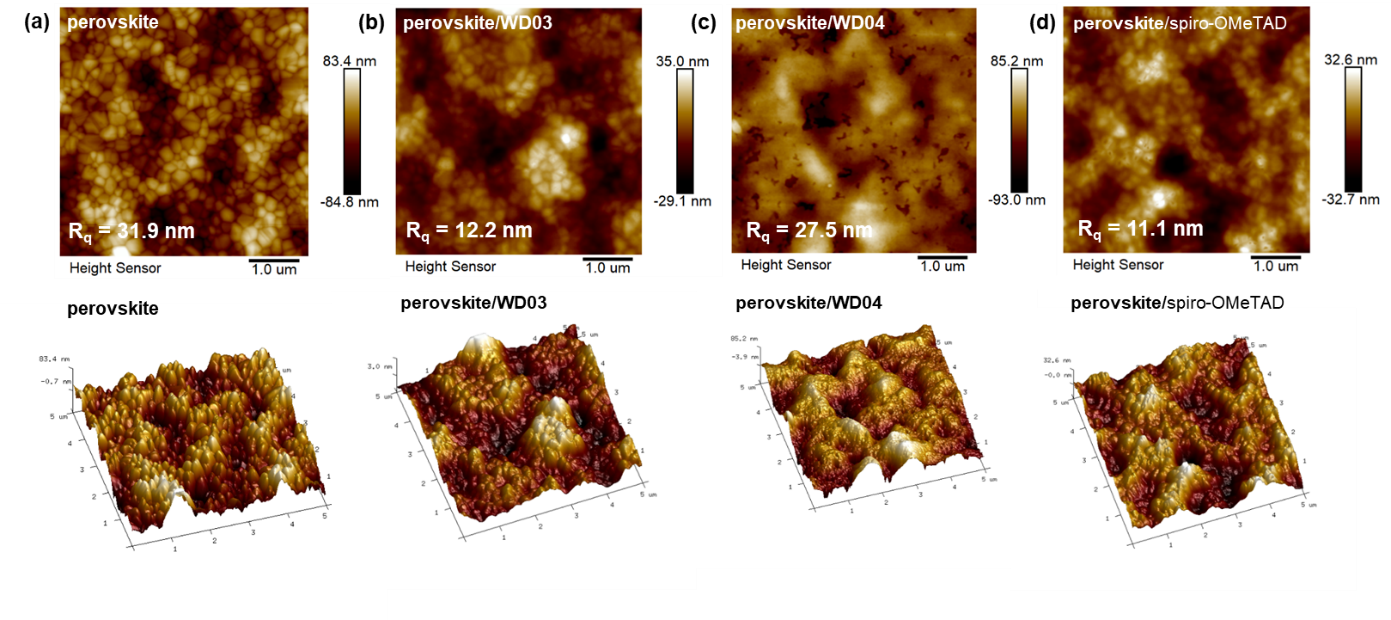


**Figure S30.** AFM height and corresponding 3D images of HTM films spin-coated on perovskite: (a) perovskite, (b) perovskite/**WD03**, (c) perovskite/**WD04**, and (d) perovskite/spiro-OMeTAD.

# **10. Fs-TA Spectra and Fitting Parameters**

**
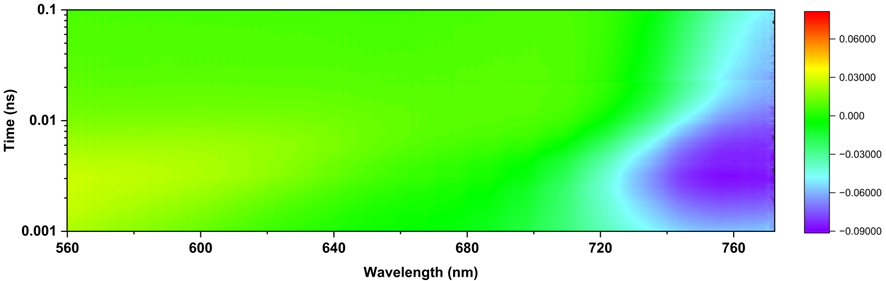
**

**Figure S31.** Fs-TA spectrum of glass/perovskite/spiro-OMeTAD film.

**
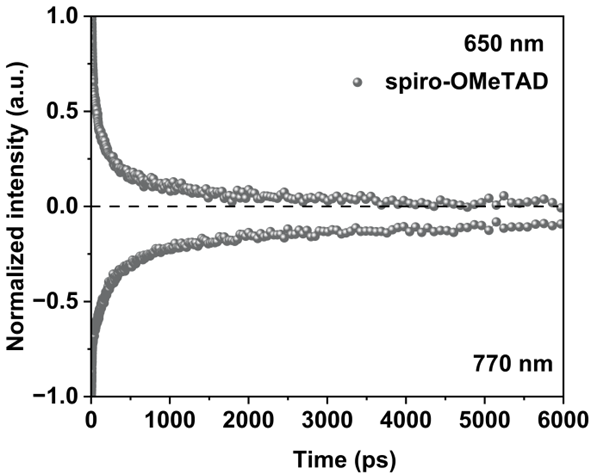
**

**Figure S32.** Normalized kinetic traces of photobleaching probed at 650 and 770 nm, respectively.

**Table S6.** Fitting parameters of bi-exponential decay function in fs-TA spectra

| sample | *λ*  (nm) | fraction  A_1_ | *τ*_1_  (ps) | fraction  A_2_ | *τ*_2_  (ps) | average decay  time *τ* (ps)^a^ |
| --- | --- | --- | --- | --- | --- | --- |
| glass/perovskite/**WD03** | 650 | 59.0% | 11.27 | 41.0% | 381.62 | 366.52 |
|  | 770 | 37.5% | 10.82 | 62.5% | 455.11 | 448.86 |
| glass/perovskite/**WD04** | 650 | 56.1% | 53.70 | 43.9% | 602.41 | 546.30 |
|  | 770 | 46.4% | 52.60 | 53.6% | 589.60 | 551.10 |
| glass/perovskite/spiro-OMeTAD | 650 | 57.5% | 19.90 | 42.5% | 333.53 | 310.10 |
|  | 770 | 45.9% | 10.13 | 54.1% | 419.24 | 411.02 |

^a^ Average decay time is calculated according to the equation: *τ* = (A_1_*τ*_1_^2^+ A_2_*τ*_2_^2^)/(A_1_*τ*_1_+ A_2_*τ*_2_).

# **11. Photovoltaic Parameters of PSCs**





**Figure S33.** *J*–*V* curve of PSC based on doped **WD04**.





**Figure S34.** IPCE spectrum and integrated current density of device based on doped **WD03**.

**Table S7.** Photovoltaic parameters of devices based on doped HTMs

| HTM | *J*_sc_ (mA cm^−2^) | *V*_oc_ (V) | FF (%) | PCE (%) |
| --- | --- | --- | --- | --- |
| **WD03** | 25.87 | 1.194 | 83.5 | 25.79 |
| **WD04** | 26.01 | 1.145 | 79.5 | 23.67 |
| spiro-OMeTAD | 26.18 | 1.179 | 83.3 | 25.71 |

**Table S8.** Photovoltaic parameters of devices based on dopant-free HTMs

| HTM | *J*_sc_ (mA cm^−2^) | *V*_oc_ (V) | FF (%) | PCE (%) |
| --- | --- | --- | --- | --- |
| **WD03** | 23.91 | 1.131 | 77.5 | 20.95 |
| **WD04** | 24.11 | 1.117 | 74.8 | 20.14 |
| **WD03**/DPB | 24.98 | 1.171 | 80.9 | 23.66 |
| spiro-OMeTAD | 24.05 | 1.106 | 76.3 | 20.29 |

**Figure S35.** Continuous maximum power point tracking of the cells containing different HTMs under conditions of N_2_ and 65 ℃.

# **References**

[1] Xia, J.; Joseph, V.; Sutanto, A. A.; Balasaravanan, R.; Ezhumalai, Y.; Zhang, Z.-X.; Ni, J.-S.; Yogesh, S. T.; Yau, S.-L.; Shao, G.; Qiu, Z.; Asiri, A. M.; Chen, M.-C.; Nazeeruddin, M. K. Isomeric Imidazole Functionalized Bithiophene-Based Hole Transporting Materials for Stable Perovskite Solar Cells. *Cell* *Rep*. *Phys*. *Sci*. **2023**, *4* (3), 101312.

[2] Frisch, M. J.; Trucks, G. W.; Schlegel, H. B.; Scuseria, G. E.; Robb, M. A.; Cheeseman, J. R.; Scalmani, G.; Barone, V.; Mennucci, B.; Petersson, G. A.; Nakatsuji, H.; Caricato, M.; Li, X.; Hratchian, H. P.; Izmaylov, A. F.; Bloino, J.; Zheng, G.; Sonnenberg, J. L.; Hada, M.; Ehara, M.; Toyota, K.; Fukuda, R.; Hasegawa, J.; Ishida, M.; Nakajima, T.; Honda, Y.; Kitao, O.; Nakai, H.; Vreven, T.; Montgomery, J. A.; Peralta, J. E., Jr.; Ogliaro, F.; Bearpark, M.; Heyd, J. J.; Brothers, E.; Kudin, K. N.; Staroverov, V. N.; Kobayashi, R.; Normand, J.; Raghavachari, K.; Rendell, A.; Burant, J. C.; Iyengar, S. S.; Tomasi, J.; Cossi, M.; Rega, N.; Millam, J. M.; Klene, M.; Knox, J. E.; Cross, J. B.; Bakken, V.; Adamo, C.; Jaramillo, J.; Gomperts, R.; Stratmann, R. E.; Yazyev, O.; Austin, A. J.; Cammi, R.; Pomelli, C.; Ochterski, J. W.; Martin, R. L.; Morokuma, K.; Zakrzewski, V. G.; Voth, G. A.; Salvador, P.; Dannenberg, J. J.; Dapprich, S.; Daniels, A. D.; Farkas, O.; Foresman, J. B.; Ortiz, J. V.; Cioslowski, J.; Fox, D. J. Gaussian 09W, Version 7.0, Gaussian, Inc., Wallingford CT, **2009**.

[3] Dennington, R. D., II; Keith, T. A.; Millam, J. M. GaussView, Version 5.0.8, Gaussian, Inc., Wallingford CT, **2008**.

[4] Xu, L.; Huang, P.; Zhang, J.; Jia, X.; Ma, Z.; Sun, Y.; Zhou, Y.; Yuan, N.-Y.; Ding, J.-N. *N*,*N*‑Di-*para*-methylthiophenylamine-Substituted (2-Ethylhexyl)‑9*H*‑carbazole: A Simple, Dopant-Free Hole-Transporting Material for Planar Perovskite Solar Cells. *J*. *Phys*. *Chem*. *C* **2017**, *121* (40), 21821–21826.

[5] Capodilupo, A. L.; Vergaro, V.; Baldassarre, F.; Cardone, A.; Corrente, G. A.; Carlucci, C.; Leporatti, S.; Papadia, P.; Gigli, G.; Ciccarella, G. Thiophene-Based Fluorescent Probes with Low Cytotoxicity and High Photostability for Lysosomes in Living Cells. *Biochim*. *Biophys*. *Acta*, *Gen*. *Subj*. **2015**, *1850* (2), 385–392.

[6] Chen, J.; Xia, J.; Yu, H.-J.; Zhong, J.-X.; Wu, X.-K.; Qin, Y.-S.; Jia, C.; She, Z.; Kuang, D.-B.; Shao, G. Asymmetric 3D Hole-Transporting Materials Based on Triphenylethylene for Perovskite Solar Cells. *Chem*. *Mater*. **2019**, *31* (15), 5431–5441.

[7] Xu, L.; Huang, P.; Zhang, J.; Jia, X.; Ma, Z.; Sun, Y.; Zhou, Y.; Yuan, N.-Y.; Ding, J.-N. N,N‑Di-para-methylthiophenylamine-Substituted (2-Ethylhexyl)‑9H‑carbazol e: A Simple, Dopant-Free Hole-Transporting Material for Planar Perovskite Solar Cells. J. Phys. Chem. C 2017, 121 (40), 21821–21826.

[8] Capodilupo, A. L.; Vergaro, V.; Baldassarre, F.; Cardone, A.; Corrente, G. A.; Carlucci, C.; Leporatti, S.; Papadia, P.; Gigli, G.; Ciccarella, G. Thiophene-Based Fluorescent Probes with Low Cytotoxicity and High Photostability for Lysosomes in Living Cells. Biochim. Biophys. Acta, Gen. Subj. 2015, 1850 (2), 385–392.

[9] Chen, J.; Xia, J.; Yu, H.-J.; Zhong, J.-X.; Wu, X.-K.; Qin, Y.-S.; Jia, C.; She, Z.; Kuang, D.-B.; Shao, G. Asymmetric 3D Hole-Transporting Materials Based on Triphenylethylene for Perovskite Solar Cells. Chem. Mater. 2019, 31 (15), 5431–5441.
